# Role of the inhibitor of serine peptidase 2 (ISP2) of *Trypanosoma brucei rhodesiense* in parasite virulence and modulation of the inflammatory responses of the host

**DOI:** 10.1371/journal.pntd.0009526

**Published:** 2021-06-21

**Authors:** David Jessula Levy, Amy Goundry, Raquel S. S. Laires, Tatiana F. R. Costa, Carlos Mendes Novo, Dennis J. Grab, Jeremy C. Mottram, Ana Paula C. A. Lima

**Affiliations:** 1 Instituto de Biofisica Carlos Chagas Filho, Universidade Federal do Rio de Janeiro, Av. Carlos Chagas Filho 373, Centro de Ciências da Saúde, Ilha do Fundão, Rio de Janeiro, RJ, Brazil; 2 Instituto de Higiene e Medicina Tropical, Universidade Nova de Lisboa, Lisboa, Portugal; 3 Wellcome Trust Centre for Molecular Parasitology, Institute of Infection, Immunity and Inflammation, University of Glasgow, Glasgow, United Kingdom; 4 Department of Pathology, Uniformed Services University of the Health Sciences, Bethesda, Maryland; Department of Pathology, Johns Hopkins University School of Medicine, Baltimore, Maryland, United States of America; 5 York Biomedical Research Institute and Department of Biology, University of York, York, United Kingdom; University of Liverpool, UNITED KINGDOM

## Abstract

*Trypanosoma brucei rhodesiense* is one of the causative agents of Human African Trypanosomiasis (HAT), known as sleeping sickness. The parasite invades the central nervous system and causes severe encephalitis that is fatal if left untreated. We have previously identified ecotin-like inhibitors of serine peptidases, named ISPs, in trypanosomatid parasitic protozoa. Here, we investigated the role of ISP2 in bloodstream form *T*. *b*. *rhodesiense*. We generated gene-deficient mutants lacking ISP2 (Δ*isp2*), which displayed a growth profile *in vitro* similar to that of wild-type (WT) parasites. C57BL/6 mice infected with Δ*isp2* displayed lower blood parasitemia, a delayed hind leg pathological phenotype and survived longer. The immune response was examined at two time-points that corresponded with two peaks of parasitemia. At 4 days, the spleens of Δ*isp2*-infected mice had a greater percentage of NOS2^+^ myeloid cells, IFN-γ^+^-NK cells and increased TNF-α compared to those infected with WT and parasites re-expressing ISP2 (Δ*isp2*:*ISP2*). By 13 days the increased NOS2^+^ population was sustained in Δ*isp2*-infected mice, along with increased percentages of monocyte-derived dendritic cells, as well as CD19^+^ B lymphocytes, and CD8^+^ and CD4^+^ T lymphocytes. Taken together, these findings indicate that ISP2 contributes to *T*. *b*. *rhodesiense* virulence in mice and attenuates the inflammatory response during early infection.

## Author summary

*Trypanosoma brucei rhodesiense* are protozoan parasites that cause deadly diseases in humans. The parasites are transmitted by the bite of infected tsetse flies and can penetrate the brain, causing severe inflammation, accompanied by motor disability, neurological disorders and death. It is important to understand how and which parasite factors contribute to infection in order to improve treatment alternatives. In their genome, these parasites have genes called *ISP*s similar to genes of bacteria, which encode proteins that inhibit serine peptidases of the trypsin family. Here, we studied the role of ISP2 in *T*. *b*. *rhodesiense* by analyzing parasite knock-out mutants for *isp2* in experimental infections in mice. We found that ISP2 contributes to blood parasitemia, to the clinical signs of motor disability, and to reduce the levels of the inflammatory response of the host, therefore, contributing to infection.

## Introduction

Human African trypanosomiasis (HAT), or sleeping sickness, is a debilitating, mostly fatal disease caused by subspecies of the protozoan parasite *Trypanosoma brucei* in the sub-Saharan region. The disease is characterized by two stages; in the first, early hemolymphatic stage, parasites spread through the bloodstream and lymph to multiple organs and to the lymph nodes [1], adipose tissue [2] and skin [3]. The second, later encephalitic stage is initiated when the parasites invade the central nervous system (CNS), which is when the most severe clinical manifestations take place. *T*. *b*. *gambiense* provokes a long-term chronic infection, while *T*. *b*. *rhodesiense* induces a fast-developing disease leading to death in a few months if left untreated [1,4]. Experimental rodent infection models with *T*. *b*. *brucei*, which is not infective to humans, have provided growing knowledge on how neuroinflammation occurs [5]. Parasite penetration and the infiltration of CD4^+^ T lymphocytes in the brain require IFN-γ, CXCL10 and TNF-α [6–8]. Myeloid cells involving the M1 phenotype associated with TNF-α are crucial to control parasite burden in the blood during early infection [9], while the B cell-related responses are central for an anti-parasite defense [10].

The biology of *T*. *b*. *brucei* has been extensively studied both genetically and at the cellular level, allowing the identification of novel potential genes and/or processes essential for parasite viability for growing axenically *in vitro* [11]. Less is known about the parasite molecules that contribute to disease development in the mammalian host. Among those, the variant surface glycoprotein (VSG) and/or its glycosylphosphatidylinositol (GPI)-anchor [12–14], and parasite CpG DNA can activate myeloid cells [15]; the parasite phospholipase C contributes to blood parasitemia and parasite lethality to mice [16]; the trypanosome lymphocyte triggering factor (TLTF) likely induces IFN-γ secretion by natural killer (NK) cells [17]; the parasite kinesin heavy chain 1 (TbKHC1) induces IL-10 and arginase-1 as well as downregulating inducible nitric oxide synthase (iNOS) activity in myeloid cells [18]; parasite adenylate cyclase is upregulated upon parasite phagocytosis by M1-type myeloid cells in the liver, inhibiting/suppressing macrophage activation [19]; and stress-response protein kinases were identified in RNAi screens as important for parasite survival in mice [20].

Ecotins are potent protein inhibitors of S1A family serine peptidases, many of which, are crucial in the mammalian response to pathogens, such as neutrophil elastase (NE), cathepsin G (CG), proteinase 3, in addition to factor Xa and XIIa, C3-convertase and plasma kallikrein [21]. We have previously identified in pathogenic protozoa, including *T*. *brucei*, genes encoding ecotin-like serine peptidase inhibitors that were named ISPs [22]. ISPs are present in *T*. *brucei*, *T*. *cruzi* and in *Leishmania* spp. in different numbers, all sharing approximately 30% sequence similarity between each other and with bacterial ecotins. *ISP1* and *ISP2* are present in *T*. *brucei* and *Leishmania* spp., while only *ISP2* could be identified in *T*. *cruzi* [22]. Recombinant *L*. *major* ISP2 inhibits NE, CG and trypsin at the nanomolar range, while ISP1 is a poor inhibitor, and plays a structural role related to the maintenance of flagellum integrity and parasite motility [23]. In a systematic study of the role of ISP2 in *L*. *major*, we found that it targets NE at the surface of macrophages during parasite phagocytosis, leading to Toll-like receptor 4 (TLR4)-mediated responses involved in the production of type I IFNs and parasite killing [24,25]. *In vivo* infection models using low parasite dose revealed that ISP2 is required for the parasite persistence in the lesion, through the dampening of the monocyte infiltration and of the NOS2 expression and IFN-γ production, providing evidence that ISP2 is a virulence factor for a cutaneous *Leishmania* species [26]. More recently, we have found that the viscerotropic *L*. *donovani* utilizes the NE-induced host type-I IFN for survival and intracellular development, and therefore has adapted to prevent ISP2 expression in order to allow successful infection [27]. To date, the role of ISP2 in *T*. *brucei* has not been addressed.

Given the potential contribution of *T*. *brucei* ISP2 (TbISP2) at the host-parasite interface, we undertook the investigation of its role using the human infective *T*. *b*. *rhodesiense*. Our results point to a crucial role of TbISP2 for parasite virulence in mice, contributing to the reduction of NOS2 expression by myeloid cells throughout infection, and to down-modulation of the generation of IFN-γ^+^ NK cells during early infection.

## Methods

### Ethics statement

All animal procedures were undertaken in adherence to experimental guidelines and procedures approved by the Ethics Committee on Animal Use of UFRJ (Comissão de Ética no Uso de Animais, CEUA) 03/15–UFRJ. All experiments were performed in accordance with the guidelines and regulations of the National Council of the Control of Animal Experimentation (Conselho Nacional de Controle de Experimentação Animal, CONCEA)—Resolução Normativa N° 39, Brasília, 20 de junho de 2018.

### Parasites

Bloodstream form (BSF) parasites of *T*. *b*. *rhodesiense* IL1852 were grown in HMI-9 medium (Gibco) supplemented with 10% fetal bovine serum (FBS) (Gibco) and 10% serum plus (JRH Biosciences), and incubated at 37°C and 5% CO_2_. Parasites were passed to fresh medium every 2 days. Antibiotic concentrations used for selection were 5 μg ml^-1^ hygromycin B (Calbiochem), 1 μg ml^-1^ G418 (Calbiochem) and 1.5 μg ml^-1^ phleomycin (InvivoGen). For growth curves, parasites were seeded in medium at a concentration of 10^4^ parasites ml^-1^ and counted daily using a Neubauer chamber on an inverted light microscope. On the third day, parasites were diluted to the initial concentration and counting continued. The cumulative cell number was calculated by multiplying by the dilution factor.

Cell lysates were extracted from 2×10^6^ parasites in the mid-log phase of growth. Parasites were centrifuged at 1500 g for 10 min, washed twice with phosphate-buffered saline (PBS), resuspended in 1× SDS-PAGE loading buffer and boiled for 5 min.

### Generation of transgenic lines

Parasite lines deficient in *ISP2* (Δ*isp2*) and re-expressing *ISP2* (Δ*isp2*:*ISP2*) were generated by the sequential gene replacement of both alleles of the wild-type (WT) gene locus and the subsequent re-introduction of the gene into the tubulin locus. Genomic DNA from *T*. *b*. *rhodesiense* 1852 WT parasites was extracted using the DNeasy Blood & Tissue kit (Qiagen). To generate the *ISP2* knock-out constructs, the 5’ and 3’ *ISP2* flanking regions were amplified from the genomic DNA by PCR using Taq DNA polymerase with primers OL2421/OL2422 for 5’*ISP2* and OL2423/OL3258 for 3’*ISP2* (Table 1). The resulting PCR products were individually sub-cloned into the pGEM-T-Easy vector (Promega), which was confirmed by sequencing. The 5’and 3’ *ISP2* flanking regions were digested from these vectors with NotI/XbaI and ApaI/XhoI, respectively, and ligated into similarly digested plasmids with hygromycin and neomycin resistance cassettes to generate the plasmids pGL1959 and pGL1960 respectively. To generate the *ISP2* re-expression construct, the *ISP2* gene was amplified from the genomic DNA using Pfu Turbo polymerase and primers OL3637/OL3638 (Table 1) and cloned into the pPCR Script vector (Stratagene). After confirming the sequence, the gene was excised from the sub-cloning vector using EcoRV and ligated into a similarly digested plasmid containing a phleomycin resistance cassette to generate the plasmid pGL2050. The plasmids, pGL1959 and pGL1960, were digested with NotI/XhoI, and the linearized DNA was gel extracted (Qiagen) and subsequently ethanol precipitated. In total, 10^7^ mid-log parasites were electroporated in 100 μl Human T Cell Nucleofector solution (Lonza) with 20 μg DNA using an Amaxxa Nucleofector. Parasites were cloned in 96-well plates in the presence of the selection antibiotics. Deletion and integration in the correct genome location of selected clones was confirmed by PCR, Southern blotting and Western blotting.

**Table 1:** Oligonucleotides used to generate constructs.

Oligo	Sequence
OL2421	TGCGGCCGCGAGCATGAATTAGGCAGAATG
OL2422	GTCTAGATCGCTTCCTTTCGCGGGTAAC
OL2423	TGGGCCCTCGTTACAACAGCCAACTACC
OL3258	CCGGGCCCCTCGAGACGCACACTGACGGCCACAC
OL3637	GCGATATCATGACAGACCGACCTCCGAC
OL3638	GCGATATCCTAACCCGCCCTCTCCTCGA

Underlined, restriction site.

### Southern blotting

For each cell line generated, approximately 5 μg of genomic DNA was digested overnight with the *Ava*I and *Bam*HI and separated by electrophoresis in 0.8% agarose gels. The gel was washed for 10 min in 0.25 M HCl, rinsed in distilled water then washed with denaturation buffer (1.5 M NaCl, 0.5 M NaOH) for 15 to 30 min before being rinsed again with distilled water. Finally, the gel was washed with neutralization buffer (3 M NaCl, 0.5 M Tris-HCl, pH 7.0) for 30 min and rinsed with distilled water. DNA was transferred overnight to a Hybond N nylon membrane (GE Healthcare) by capillary force in 20× SSC buffer (3 M NaCl, 0.3 M sodium citrate, pH 7.0). After the transfer, the membrane was washed for 10 min in 2× SSC buffer and the DNA was crosslinked to the membrane in a UV Stratalinker 2400 crosslinker (Stratagene) at 1200 mJ. For the probing, the Gene Images Alk-Phos Direct Labelling and Detection System (GE Healthcare) was used, according to the manual, to generate a fluorescent-labeled DNA probe. The 5’ flanking region of ISP2, digested out of the respective plasmid and purified from agarose gel, was used as a probe. Signal was detected using CDP-Star detection reagent (GE Healthcare).

### Production and purification of monoclonal antibodies and recombinant protein

For the generation of monoclonal antibodies against *T*. *b*. *rhodesiense* ISP2, two female BALB/c mice were immunized with 10 μg of recombinant TbISP (rTbISP) intraperitoneally every 2 weeks. For the production of rTbISP1 and rTbISP2, *T*. *brucei ISP1* and *ISP2* were amplified by PCR from the genomic DNA of *T*. *b*. *brucei* WT parasites and ligated into similarly digested pET3a (Novagen). The constructs pGL1057 and pGL1059 were then used to express C-terminal his-tagged ISP1 and ISP2 respectively. The proteins were isolated using nickel agarose affinity purification, ion-exchange chromatography. Lipopolysaccharide (LPS) was removed using Detoxi-Gel (Pierce) according to manufacturer’s instructions.

Cell fusions were performed as previously described. Briefly, hypoxanthine-aminopterin-thymidine (HAT)-sensitive Sp2/0 myeloma cells and the spleen cells of mice immunized four times were performed at a 10:1 ratio by co-centrifugation at 1200 rpm for 10 min in the presence of polyethylene glycol (PEG). The cell pellet was resuspended in PEG-DMSO Hybri-Max (Sigma-Aldrich) and subsequently grown in Dulbecco´s Modified Eagle Medium (DMEM) (Gibco) supplemented with 10% FBS and 1x HAT (Sigma-Aldrich), plated in 96-well plates and incubated at 37°C, 5% CO_2_ and 95% relative humidity. After 10 days, wells were observed under microscope for the presence of hybridomas, which were then tested by ELISA using rTbISP as the antigen. Positive clones were selected and grown in culture flasks for 21–28 days. Cultures were centrifuged at 1200 rpm for 10 min, and the supernatant was filtered through a 45 μm filter and dialyzed in a dialysis membrane immersed in binding buffer (20 mM sodium phosphate, pH 7.0) at 4°C overnight, which was subsequently affinity purified using a HiTrap Protein G HP column (GE Healthcare). Purified fractions were pooled, concentrated with 4 M ammonium sulfate and dialyzed against PBS at 4°C overnight. Purified antibodies were filter-sterilized and stored at 4°C until use.

### Western blotting

Samples of recombinant TbISP (100 ng) or whole cell lysate (5x10^6^ BSF parasites) were separated on a 15% SDS-PAGE gel and transferred to a Hybond C nitrocellulose membrane (GE Healthcare). The membrane was blocked with Tris-buffered saline (TBS) with Tween 20 [25 mM Tris, 150 mM NaCl, 2 mM KCl, 0.1% Tween 20] and 5% w/v milk powder (Marvel) for 1–2 h at room temperature. Anti-TbISP2 was used at 1:500 and incubated for 1–2 h at room temperature. Membranes were washed three times for 5 min each then incubated with an anti-mouse IgG HRP antibody (Promega) at 1:10,000 for 1–2 h at room temperature. The signal was developed using SuperSignal West Pico Chemiluminescent Substrate (Pierce ThermoScientific).

Densitometry was calculated by determining the areas of the detected bands using the ImageJ software (U.S.A National Institutes of Health, Bethesda, MD, USA). The areas were normalized to the respective loading controls by the ratio between sample/loading control. To assess any change between the different samples, the normalized areas of bands for the WT line were considered as 100% (= 1) and the normalized areas of the other lines were then calculated as percentages in relation to the WT.

### Immunofluorescence

BSF *T*. *b*. *rhodesiense* in the mid-log phase of growth were washed in Voorhei’s modified PBS (vPBS) [137 mM NaCl, 3 mM KCl, 16 mM Na_2_HPO_4_, 3 mM KH_2_PO_4_, 46 mM sucrose, 10 mM glucose, pH 7.6] and fixed in 3% paraformaldehyde for 10 min then washed twice with vPBS. Parasites were applied to poly-L-lysine-coated slides for 20 min and permeabilized with vPBS/0.1% Triton X-100 for 10 min. Parasites were washed three times with vPBS then blocked for 1 h with vPBS/20% FBS. Parasites were incubated with anti-TbISP2 antibodies diluted at 1:25 at 4°C overnight. Slides were washed three times with vPBS then an anti-mouse Alexa Fluor 594 antibody (Molecular Probes) was used at 1:4,000 for 1 h at room temperature. Slides were washed three times with PBS and stained with 1 μg ml^-1^ DAPI in mounting solution [PBS, 50% (v/v) glycerol, 2.5% (w/v) DABCO] before being sealed with a coverslip.

### Mice

Female C57BL/6J mice between 10 and 18 weeks were obtained from the in-house breeding facility at UFRJ. Mice were infected intraperitoneally with 1×10^5^ *T*. *brucei rhodesiense* bloodstream form in 100 μl RPMI. Parasitemia was determined through a minimal incision at the tip of the tail and manual pressure until a drop of blood appeared and 5 μl could be taken and passed to a slide. A coverslip was added onto the blood drop and the parasites were counted on a light microscope and parasitemia was calculated according to the Brener protocol.

### Study of motor activity

The back paws of mice were lightly pressed in non-toxic blue ink. The mice were then put at the start of a 30 cm track with filter paper underneath and allowed to walk the entire distance. The distance between one footstep and the next was measured and repeated for at least 4 footsteps.

### Tissue processing

On the 4^th^ or 13^th^ day post-infection, mice were euthanized and perfused with 0.9% NaCl. The spleens and livers were them removed and the masses were recorded. Spleens were macerated through nylon in 800 μl RPMI containing a protease inhibitor cocktail (Sigma-Aldrich). Livers were macerated through 70 μM cell strainers. The cell suspensions were centrifuged at 1500 rpm for 5 min and the spleen supernatants were collected for cytokine analysis. The cell pellets were resuspended in ACK for 1 min [0.15 M NH_4_Cl, 1 M KHCO_3_, 0.1 M EDTA, pH 7.2] then 5 ml RPMI was added. The cells were centrifuged at 1500 rpm for 5 min, washed three times in RPMI then counted to obtain the total number of cells. Spleen cells were then stained and assessed by flow cytometry, while 800 μl per well of the liver homogenate after normalization to organ weight was plated and incubated at 37°C and 5% CO_2_. After 24 h, plates were centrifuged at 1500 rpm for 5 min and the supernatants were collected for cytokine analysis.

### Flow cytometry

All steps were performed at 4°C and all centrifugation steps were performed at 1500 rpm for 5 min. For extracellular staining, single-cell suspensions from the spleen at 6×10^6^ cells were washed in PBS and incubated with an anti-Fc-γ III/II (CD16/32) receptor antibody (BioLegend) for 10 min. Cells were washed again in PBS then stained with the fluorochrome-conjugated antibodies for 30 min. The following anti-mouse antibodies were used: **Group 1—**FITC-CD4 (eBioscience), PE-Cy5-CD8a (eBioscience), APC-CD19 (eBioscience), and BV421-NK1.1 (BioLegend); **Group 2—**PE-CD11b (eBioscience), FITC-F4/80 (BioLegend), PerCP-Cy5.5-CD11c (BioLegend), BV605-Ly-6C (BioLegend), and APC- Ly-6G (BioLegend). For staining of cytoplasmic intracellular antigens, single-cell suspensions from the spleen at 6×10^6^ cells were resuspended in RPMI/10% FBS and incubated with 5 μM monensin (Sigma) for 4 h at 37°C and 5% CO_2_. Cells were then centrifuged and the pellet was incubated with an anti-Fc-γ III/II (CD16/32) receptor antibody (BioLegend) for 10 min. Cells were washed in PBS then stained with the fluorochrome-conjugated antibodies for 30 min. The following anti-mouse antibodies were used: **Group 3** BV421-NK1.1 (BioLegend); **Group 4—**FITC-CD11b (eBioscience), BV605-Ly-6C (BioLegend), APC-Ly-6G (BioLegend). Cells were washed in PBS then resuspended in 100 μl IC Fixation Buffer (eBioscience), incubated for 30 min, after which 500 μl Permeabilization Buffer (eBioscience) was added. The cells were centrifuged and incubated with anti-IFN-γ (APC, BioLegend) and anti-NOS2 (PE, eBioscience) in 50 μl Permeabilization Buffer to **Group 3** and **Group 4** for 30 min, respectively. Cells were washed in 500 μl Permeabilization Buffer then washed in PBS.

All groups were resuspended in 300 μl PBS and 20,000 events were acquired on a BD FACSCanto II. Data were analyzed on FlowJo v10. Gating strategies are described in figure legends and the dot plots are given as supplementary figures.

### ELISAs

The cytokine concentrations of CXCL1, IL-6, IL-12p40, TNF-α, IFN-γ and CCL2 in supernatants from the spleen *ex vivo* and from hepatocytes cultivated for 24 h were evaluated using Duoset kits specific for mouse cytokines from R&D Systems following the manufacturer’s instructions. Plates were read on an Asys Expert Plus microplate reader.

### Statistical analysis

Graphs were generated in the GraphPad Prism 5 software (GraphPad Software Inc) and the distribution of data was checked using a Kolmogorov-Smirnoff test and variance was determined by the F-value. The appropriate statistical tests were then applied as described in the figure legends.

## Results

**Fig 1:**
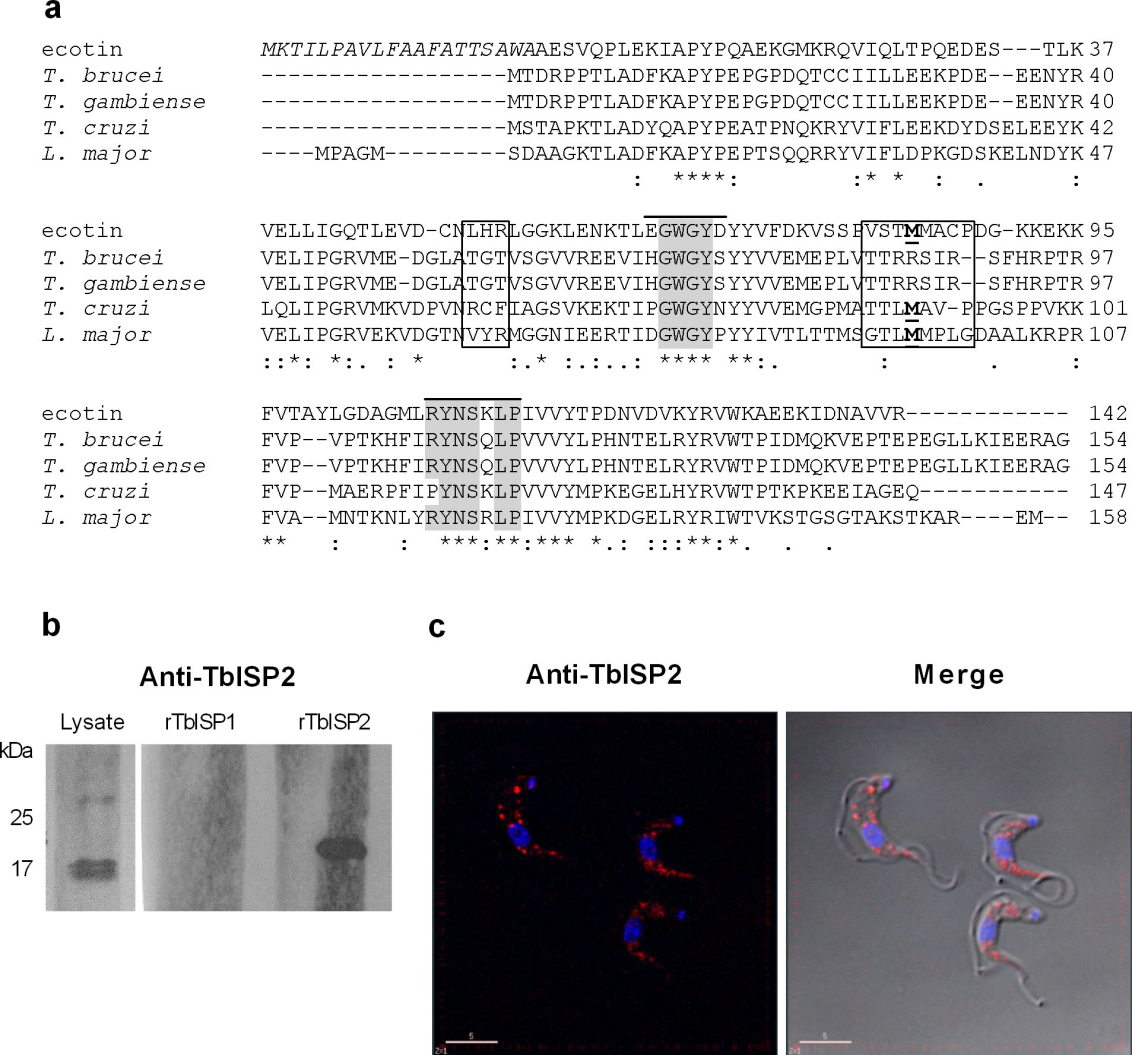
Expression of TbISP2 by *T*. *b*. *rhodesiense* bloodstream forms. (a) Alignment of predicted amino acid sequences of ecotin and ISP2. The numbering for ecotin indicates the mature protein, which starts at Ala1, and the signal peptide prior to this is given in italics. The primary enzyme recognition site (50s and 80s loops, respectively) deduced from ecotin-trypsin complex is marked in boxes and the reactive-P1 Met84 is bold and underlined. The box indicating the 80s loop of ecotin shows residues P4 to P4’ of the inhibitor’s reactive site [28]. Regions forming the secondary binding site (60s and 100s loops, respectively) in the ecotin-trypsin complex are indicated by a line at the top and the conserved residues in those regions are highlighted in grey. Ecotin: *E*. *coli* (K12) (UniProtKB—P23827 (ECOT_ECOLI), *T*. *b*. *brucei* (Tb927.5.1730), *T*. *b*. *gambiense* (Tbg972.5.2420), *T*. *cruzi* Dm28 (BCY84_00539-t36), *L*. *major* Friedlin (LmjF.15.0510). (b) Monoclonal antibodies against *T*. *brucei* ISP2 tested against *T*. *b*. *rhodesiense* whole cell lysate (lane 1), rTbISP1 100 ng (lane 2) and rTbISP2 100 ng (lane 3). (c) *T*. *b*. *rhodesiense* BSF parasites were harvested, washed in vPBS then fixed in 3% paraformaldehyde. Cells were then applied to poly-L-lysine-treated slides and permeabilized. Slides were blocked and subsequently incubated with anti-ISP2 antibodies. Anti-mouse Alexa Fluor 594 was used as secondary antibody and visualized on a rhodamine filter. Nuclear and kinetoplast DNA was stained with DAPI. Scale bar 5 μm.

In the available genomes of *T*. *brucei* subspecies *T*. *b*. *brucei* and *T*. *b*. *gambiense*, there are two ecotin-like genes located on chromosome 5. The predicted amino acid sequences of *T*. *b*. *brucei* ISP1 (Tb427_050023100) and *T*. *b*. *gambiense* ISP1 (Tbg972.5.2420) are 99% identical, sharing 54% identity with *L*. *major* ISP1. *T*. *b*. *brucei* ISP2 (Tb427_050025100) and *T*. *b*. *gambiense* ISP2 (Tbg972.5.2580) are identical and share 63% identity with *L*. *major* ISP2 (Fig 1A). Therefore, these genes were considered *ISP1* and *ISP2* orthologues, which are separated from each other by 31 kb in the genome of *T*. *b*. *gambiense*. The reactive site methionine of ecotin (Met84), which interacts with the active site of trypsin [28], is present in the equivalent position in the ISP2 of *L*. *major* (Met95) and *T*. *cruzi* (Met90) but not in *T*. *brucei* (Arg87) (Fig 1A). Mutagenesis studies with ecotin have shown that Met84 is not essential for its inhibitory activity against trypsin, but it reduced its inhibitory activity against elastase, while not affecting specificity of ecotin toeither enzyme. The authors showed that Met84 could be replaced by Ile, Arg, Glu or Tyr with little to no effect for trypsin inhibition [29]. The genome of *T*. *b*. *rhodesiense* 1852 is not available, but given the identity between the ISP genes among the other *T*. *brucei* subspecies, it is likely that the ISP proteins in *T*. *b*. *rhodesiense* will be identical to those of *T*. *b*. *brucei*.

To produce recombinant TbISPs, the *ISP1* and *ISP2* genes were generated by PCR using genomic DNA of *T*. *b*. *brucei* as a template. Histidine-tagged recombinant TbISP1 and TbISP2 were produced in *E*. *coli* and used for the production of mouse monoclonal antibodies. We produced monoclonal antibodies to TbISP2 that could confidently discriminate between ISP1 and ISP2 in *T*. *b*. *rhodesiense* BSF parasite lysates (Fig 1B). The specificity of the monoclonal antibody to TbISP2 (Fig 1B) was confirmed by Western blotting using both the recombinant TbISPs, as no cross-reactivity was observed. The predicted molecular mass of ISP2 of *T*. *b*. *brucei* is 17.8 kDa, but is slightly higher in the recombinant TbISP2 due to the presence of the His-tag. The monoclonal antibody reacted with a major protein of approximately 17 kDa in the lysate of *T*. *b*. *rhodesiense* BSF parasites (Fig 1B, lane 1), suggesting that TbISP2 is expressed in this life-stage of *T*. *brucei*. Next, we used the monoclonal antibody that was previously affinity purified against immobilized recombinant TbISP2 to determine the subcellular localization of TbISP2 in *T*. *b*. *rhodesiense* bloodstream forms by immunofluorescence (Fig 1C), in which a punctate cytoplasmic distribution for ISP2 was observed.

**Fig 2:**
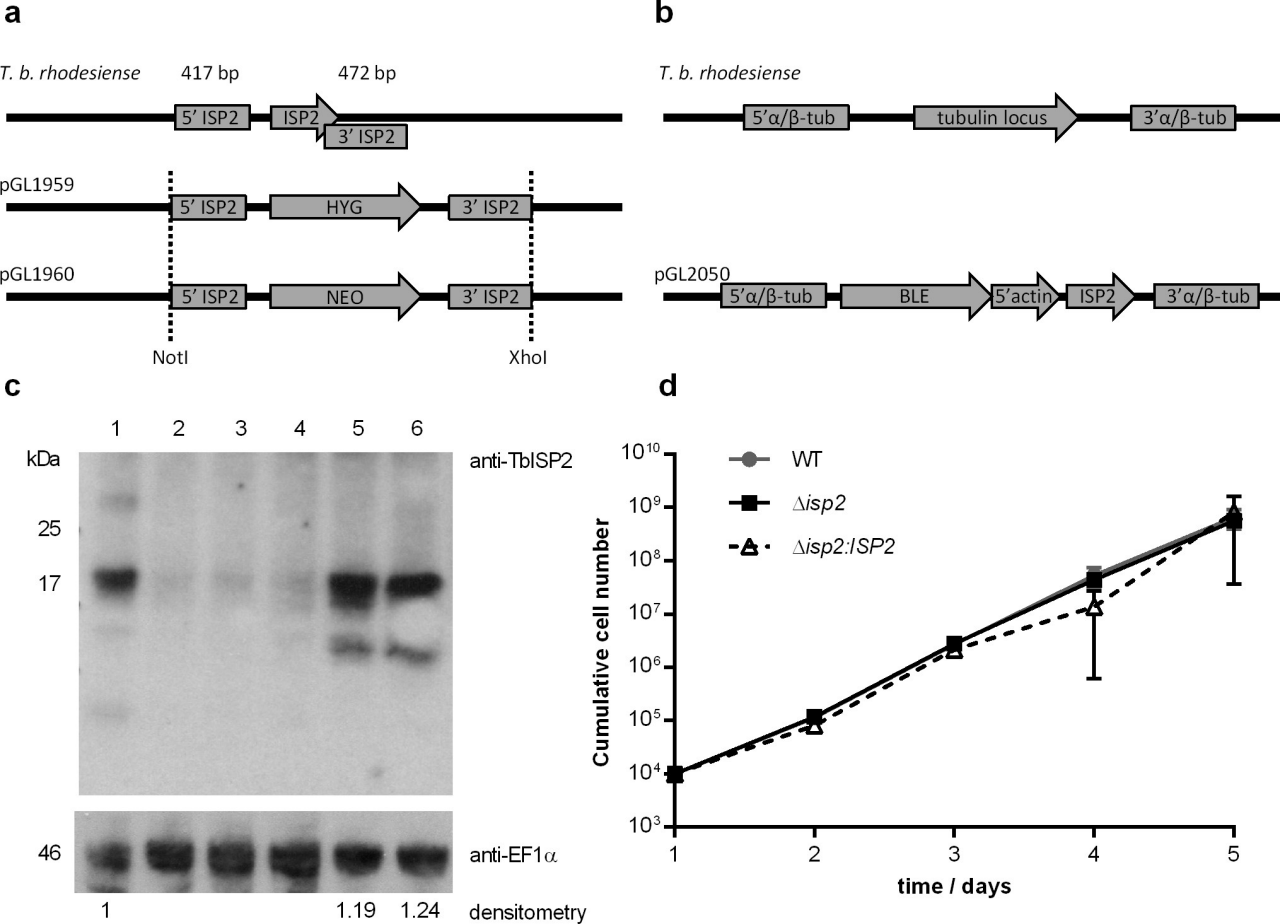
Generation of *T*. *b*. *rhodesiense* null mutants for TbISP2. (a) Schematic representation of the *ISP2* locus of *T*. *brucei* before and after integration of hygromycin (HYG) and neomycin (NEO) resistance cassettes by homologous recombination using sequences containing the 5`and 3`flanking regions of Tb*ISP2*, generating *T*. *b*. *rhodesiense* Δ*isp2*. (b) Schematic representation of *ISP2* reintegration into the tubulin locus of *T*. *b*. *rhodesiense* Δ*isp2* parasites by homologous recombination using a cassette containing the coding sequence of Tb*ISP2* flanked by fragments of the intergenic region of the *T*. *brucei* tubulin locus and a phleomycin (BLE) resistance gene, generating Δ*isp2*:*ISP2*. (c) Whole cell lysates were obtained from 5×10^6^ parasites and analyzed by Western blotting with the anti-ISP2 antibody to assess protein expression in the different cell lines. An anti-EF1α antibody was used as a loading control. Lane 1 –WT *T*. *b*. *rhodesiense* IL1852; Lanes 2, 3 and 4 –*T*. *b*. *rhodesiense* Δ*isp2* clones 1, 2 and 3; Lanes 5 and 6 –*T*. *b*. *rhodesiense* Δ*isp2*:*ISP2* clones 1 and 2. Levels of ISP2 were calculated by densitometry in relation to WT and normalized to EF1α. In lanes 5 and 6, the lower molecular mass bands were not considered in the densitometry. (d) Growth curves of BSF *T*. *b*. *rhodesiense* WT, Δ*isp2* and Δ*isp2*:*ISP2* determined by cell counts on a hemocytometer after cells were seeded at 1×10^4^ cells ml^-1^. Cultures were diluted to 1×10^4^ cells/ml every 48 h and the cumulative cell number was determined using the dilution factor. Data are represented as the mean ± SD. Experiment was performed in triplicate and graph is representative of 3 independent experiments. Statistical analysis was performed using Two Way ANOVA with Bonferroni posttest.

To address the role of ISP2, we generated *T*. *b*. *rhodesiense* bloodstream form *ISP2*-null mutant lines (Δ*isp2*) by homologous recombination (Fig 2). Recombination cassettes containing the 5`and 3`UTR sequences of Tb*ISP2* flanking drug resistance genes were constructed and transfected into *T*. *b*. *rhodesiense* (Fig 2A), followed by analysis of drug-resistant clones to identify null mutants. Southern blot analysis of the mutants confirmed the integration of drug-resistance cassettes in the Tb*ISP2* locus (S1 Fig). Two Δ*isp2* clones were subsequently transfected with a recombination cassette containing the coding sequence of Tb*ISP2* flanked by fragments of the intergenic region of the tubulin locus of *T*. *b*. *brucei* and a drug resistance gene (Fig 2B) for the generation of TbISP2 re-expressing lines (Δ*isp2*:*ISP2*). Western blotting of parasite lysates confirmed the absence of the ISP2 protein in the null mutant lines (Fig 2C, lanes 2–4) and the expression of TbISP2 in the re-expressing lines (Fig 2C, lanes 5–6). Densitometry, followed by normalization against loading controls, revealed that Δ*isp2*:*ISP2* had approximately 20% more ISP2 than WT parasites. A lower molecular mass band was detected by anti-ISP2 in the lysates of the re-expressors, possibly reflecting degradation/processing of ISP2 in those lines. There were no gross detectable morphological defects in the null mutants, and they were viable in culture. The growth rate *in vitro* was assessed by daily quantification over a 5-day period, and there were no significant differences as compared to wild-type (WT) parasites (Fig 2D).

**Fig 3:**
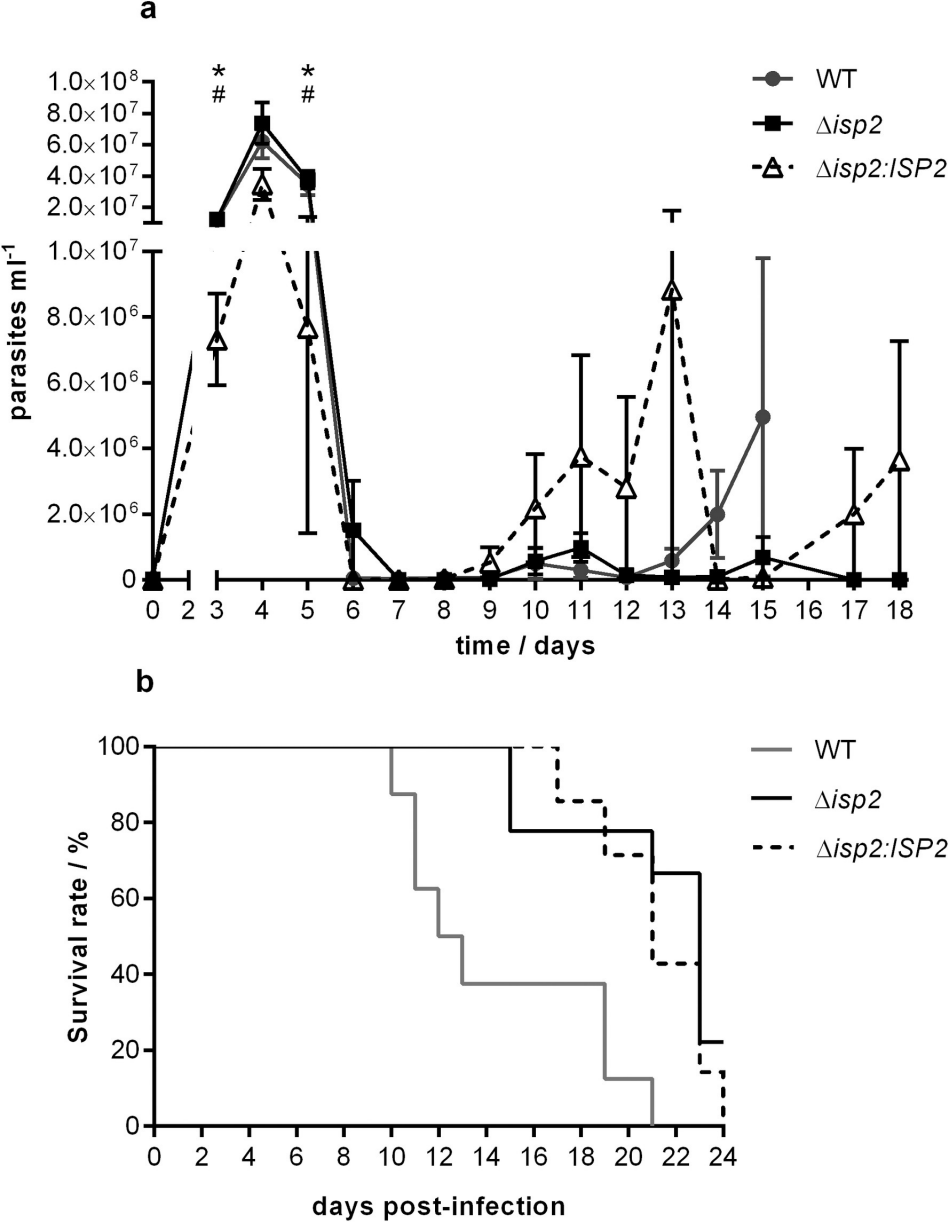
TbISP2 contributes to parasite virulence in mice. C57BL/6 mice were infected intraperitoneally with 1×10^5^ *T*. *b*. *rhodesiense* WT, Δ*isp*2 and Δ*isp*2:*ISP*2. (a) Parasitemia was calculated by counting the parasites in blood taken from the tail vein every day by microscopy from day 3 to day 18. Data are represented as the mean ± SEM (WT, n = 6 per group; Δ*isp*2, n = 5 per group; and Δ*isp*2:*ISP*2, n = 6 per group).and are representative of three independent experiments. Statistical analysis was performed using Two Way ANOVA with Bonferroni post test. * p <0.05 between WT and Δ*isp*2:*ISP*2, and # p< 0.05 between Δ*isp*2 and Δ*isp*2:*ISP*2. (b) Survival curve of infected mice (n = 7–9 per group). Graph is representative of two independent experiments.

To address whether ISP2 would influence the host response, we sought to establish a mouse model for infection with *T*. *b*. *rhodesiense*. We initially observed that C57BL/6 mice infected with WT *T*. *b*. *rhodesiense* developed visible signs of motor disability after one week, and at least two detectable waves of blood parasitemia. We then adopted this model to evaluate *in vivo* infection by the different parasite lines. Elevated blood parasitemia was observed at day 4 post-infection, which was rapidly controlled by day 6 in mice infected with all the parasite lines (Fig 3A). A second wave with 10-fold lower parasitemia started to appear at day 9 post-infection for Δ*isp2* and Δ*isp2*:*ISP2*-infected mice, and at day 13 post-infection for WT-infected mice, peaking at day15, with a similar parasite load for mice infected with WT, and Δ*isp2*:*ISP2* at days 15 and 13, respectively (Fig 3A). At day 15, mice infected with WT parasites displayed signs of prostration and motor disability in the rear legs, and were euthanized. The parasitemia was controlled by mice infected with Δ*isp2* or Δ*isp2*:*ISP2*, and in mice infected with Δ*isp2*:*ISP2*, a third wave of parasitemia appeared around day 17. In contrast, mice infected with Δ*isp2* did not show detectable parasites in the blood, suggesting that ISP2 is important for parasite fitness in the mammalian host. Lack of ISP2 also had an impact in the survival of infected mice (Fig 3B). At day 13, 63% of mice infected with WT parasites died, while all mice infected with Δ*isp2* were alive (Fig 3B). Infections with WT *T*. *b*. *rhodesiense* caused the death of all mice in 21 days of infection while mice infected with the re-expressing line Δ*isp*2:*ISP*2 had mildly extended survival, with 42% still alive at day 21, and 86% dead at day 23. In contrast, 66% of mice with the ISP2-null line, Δ*isp2*, were alive and showed mild motor irregularities at day 21. The results suggest that in the absence of ISP2, mice were more capable of controlling parasite load in the blood, which remained below the detection limit for longer periods of time, with prolonged host survival.

**Fig 4:**
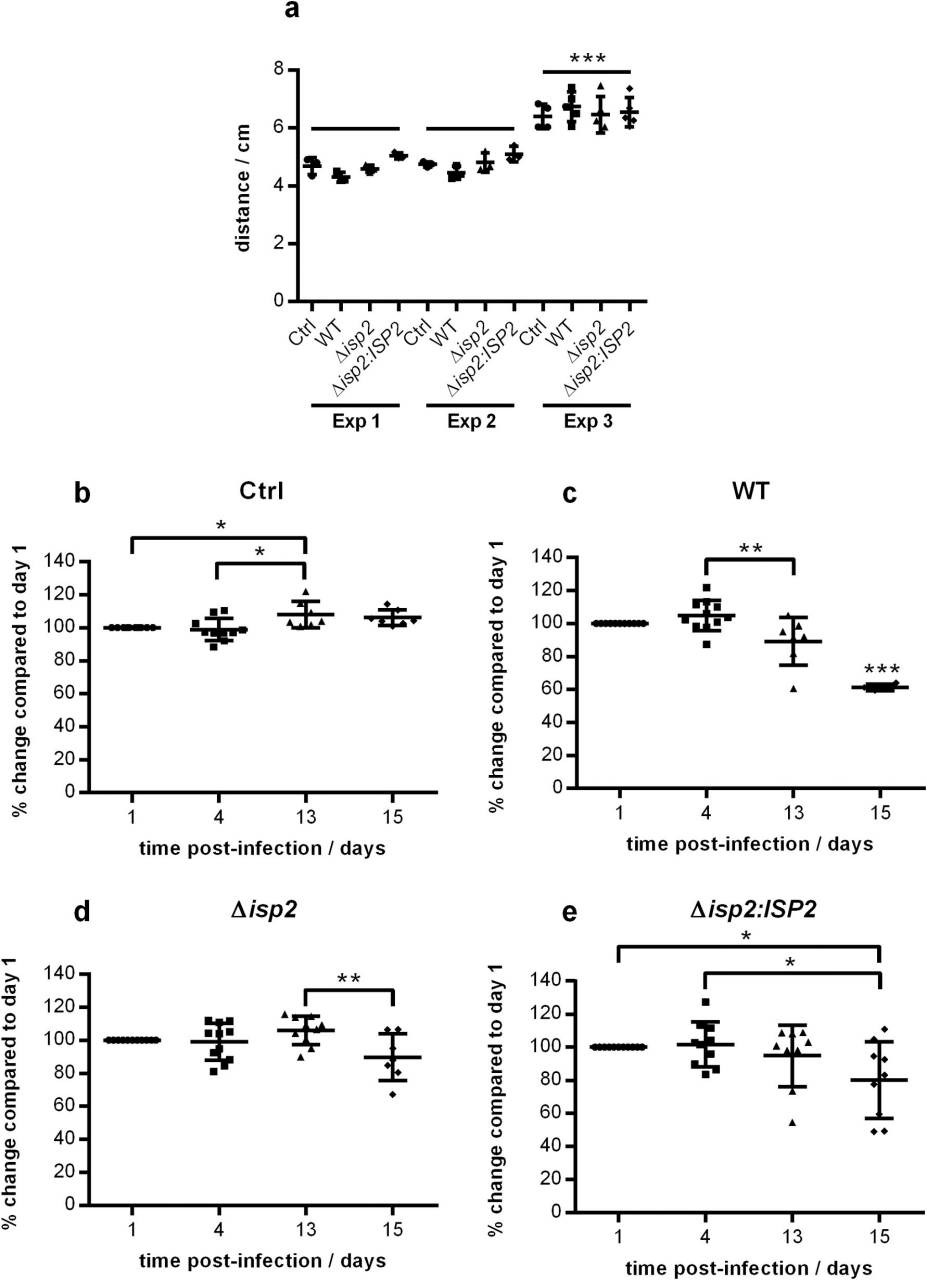
Progression of motor disorder in mice infected with *T*. *b*. *rhodesiense*. Role of TbISP2 in disease progression. C57BL/6 mice were infected intraperitoneally with 1×10^5^ *T*. *b*. *rhodesiense* WT, Δ*isp*2 and Δ*isp*2:*ISP*2. Uninfected mice were used as a control (Ctrl). Motor function was assessed by inking the back paws of the mice and placing them on a platform covered with filter paper and allowing them to walk forward. The average distance between footprints was measured for each mouse. (a) The step distance on the first day post-infection for three independent experiments. Each point represents an individual mouse. The mouse groups of experiment 3 were bigger than any of the mouse groups of the other two experiments (p <0.001). Data from the 3 experiments were combined and the % change in the step distance on day 4, 13 and 15 was calculated as compared to day 1. Graphs of the step distance changes for (b) Ctrl mice, and mice infected with (c) WT, (d) Δ*isp2* and (e) Δ*isp2*:*ISP2*. Lines indicate the mean ± SD. Statistical analysis was performed using One Way ANOVA with Tukey multiple comparison test between the time-points for each mouse group individually. * p <0.05, ** p <0.01 and *** p <0.001.

Next, we evaluated the appearance of clinical signs of motor disability as a way to address disease progression. To this end, we determined the average distance between the walking footprints of mice, as a parameter to evaluate their motor capability, at three time-points over the infection period in three independent experiments (Fig 4). At day 1 post-infection, control and infected mice displayed minimal variation of the distances between the footsteps for individual mice within the same group (Fig 4A). As the absolute values of the distances (in cm) varied among independent experiments, the distances of the footsteps at the later time points were normalized to their percentage in relation to day 1 for each individual mouse, and the normalized data of the three experiments were grouped together (Fig 4B–4E). In control mice, the distances between footsteps remained very homogenous over the time-course, with a slight increase after day 13, likely due to the growth of the animals (Fig 4B). In contrast, infected animals showed greater variability in the distances between footsteps as of day 4, until day 15 (Fig 4C–4E). Mice infected with WT *T*. *b*. *rhodesiense* displayed a significant reduction of the distance between footsteps at day 13, which decreased further at day 15 among the surviving mice, being about 60% of the distance as compared to day 1 (Fig 4C). In contrast, the distances between footsteps of mice infected with Δ*isp*2 parasites were unchanged at day 13 post-infection, and at day 15, were still kept at approximately 90% to that displayed at day 1, revealing a significant delay in the progression of motor disability-related signs (Fig 4D). Mice infected with Δ*isp*2:*ISP*2 showed large variability among individuals, and at day 15, displayed an average of 80% of the distance seen at day 1 (Fig 4E). Noteworthy, 33% of the mice displayed less than 60% of the distance as compared to day 1, resembling the behavior observed in mice infected with WT parasites, which suggests that there is partial complementation of the phenotype by the re-expressor line. Collectively, our findings indicate that although Δ*isp*2 provokes initial blood parasitemia similarly to WT parasites, the course of the infection changes significantly after the control of the first wave, resulting in lower parasite burden, delayed motor dysfunction and increased survival, indicating that TbISP2 plays a role as a virulence factor for *T*. *b*. *rhodesiense*.

**Fig 5:**
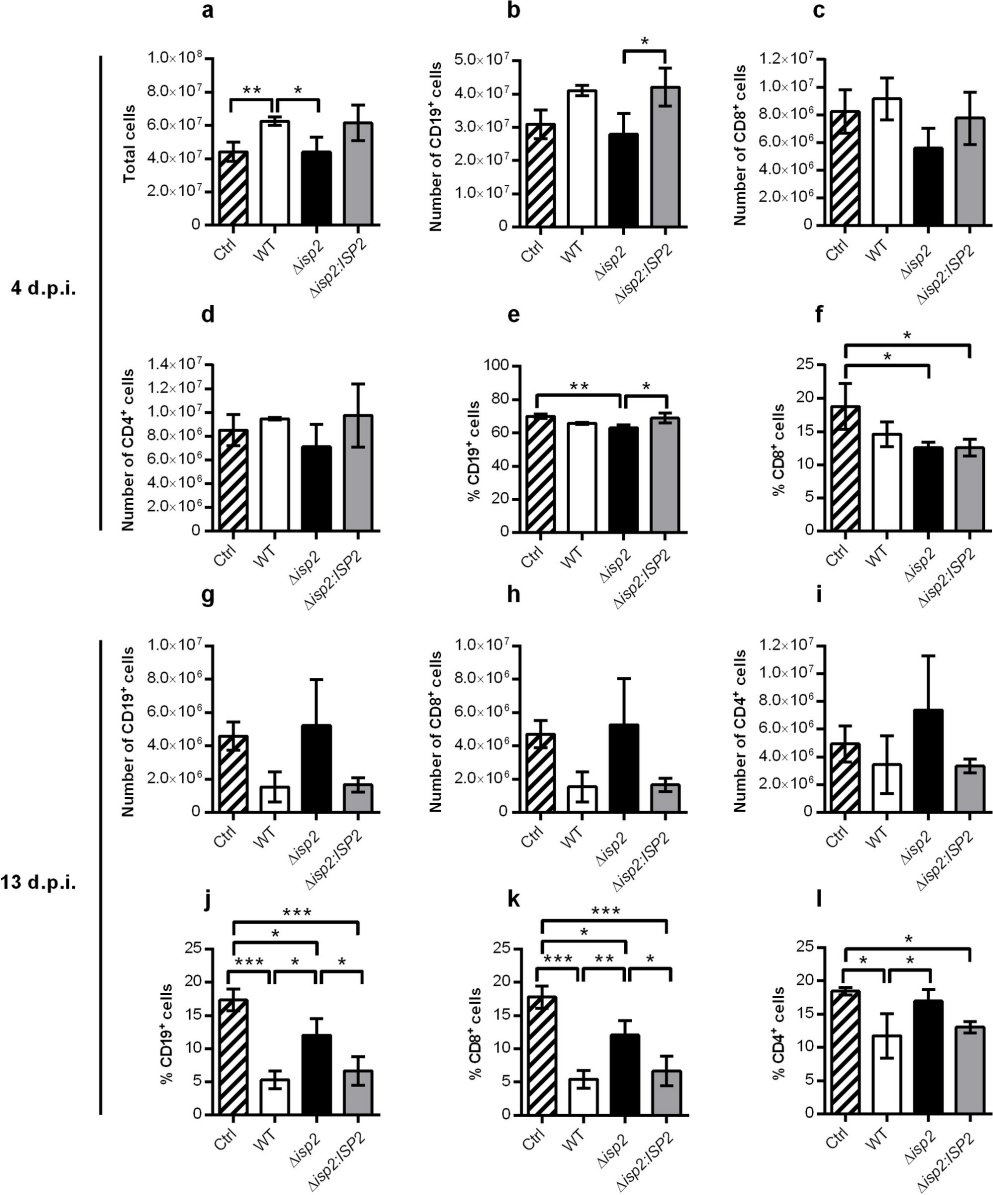
Distribution of spleen lymphocyte populations during early and late infection. C57BL/6 mice were infected intraperitoneally with 1×10^5^ *T*. *b*. *rhodesiense* WT, Δ*isp*2 and Δ*isp*2:*ISP*2. Uninfected mice were used as a control (Ctrl). After 4 days (top panel) or 13 days (bottom panel), mice were euthanized, perfused, and the spleens were removed and homogenized. Cell suspensions were counted to give (a) the total cells per spleen, and then stained with antibodies for extracellular markers for flow cytometry. Cells were first gated to exclude debris using the FSC vs. SSC plot followed by FSC-H vs. FSC-A to exclude doublets. CD19^+^ cells were gated using a SSC vs. APC-CD19 plot, while CD4^+^ and CD8^+^ cells were gated on a FITC-CD4 vs. PE-Cy5-CD8a plot. Representative plots are shown in S2 Fig. Graphs show the number and percentage of (b and e) CD19^+^ cells and (c and f) CD8^+^ cells, and (d) the number of CD4^+^ cells within the spleen at 4 days post-infection. At 13 days post-infection, graphs show the number and percentages of (g and j) CD19^+^ cells, (h and k) CD8^+^ cells, and (i and l) CD4^+^ cells. n = 3 mice per group. In (g) to (l), n = 4 mice in the group infected with Δ*isp*2. Results are presented as the mean ± SD. Data are representative of 3 independent experiments. Statistical analysis was performed using One Way ANOVA with Bonferroni posttest. * p <0.05, ** p <0.01 and *** p<0.001.

We asked if the host response mounted during the early phase of infection could contribute to the subsequent control of parasite burden. To this end, we evaluated the status of the immune response at two stages of the infection; in the early stage at the first parasitemia peak (day 4), and in the later stage at the second parasitemia peak, when the clinical signs of motor disability became more evident (day 13). First, we analyzed the cellular populations in the spleen. At day 4, the number of cells in the spleens of mice infected with WT or Δ*isp2*:*ISP2* had increased as compared to uninfected control mice (Fig 5A), with main increases in the number of CD19^+^ B lymphocytes (by 40%) (Fig 5B), while the numbers of CD8^+^ (Fig 5C) and CD4^+^ T lymphocytes (Fig 5D) remained similar to those of control mice. However, we did not observe this increase in Δ*isp2*-infected mice, which had a total cell number in the spleen similar to that of uninfected mice and this pattern was generally maintained when the different cell types were analyzed. The population of CD8^+^ T cells seemed reduced by 30% in mice infected with Δ*isp2*, albeit not statistically significant (Fig 5C). The frequencies of CD19^+^ B lymphocytes (Fig 5E) and CD8^+^ T cells (Fig 5F) were slightly reduced in infected mice as compared to control mice, while those of CD4^+^ T cells were unchanged.

At day 13 we observed an inverted pattern; i.e., the total numbers of CD19^+^ B lymphocytes (Fig 5G), CD8^+^ (Fig 5H) and CD4^+^ T lymphocytes (Fig 5I) were reduced in infected animals, as compared to control mice. The variability among individual mice in the groups precluded the observance of statistical significance. The frequencies of these three lymphocyte subtypes were markedly reduced in infected mice as compared to control mice (Fig 5J–5L). The frequencies of CD8^+^ T cells (Fig 5K) and CD4^+^ T cells (Fig 5L) in Δ*isp2*-infected mice were closer to those of control mice. Of potential relevance, there was a striking depletion of CD19^+^ B cells in mice infected with WT and Δ*isp*2:*ISP*2, while mice infected with Δ*isp*2 had approximately 3-fold higher frequency of those cells (Fig 5J), associating the lack of TbISP2 presumably with protection of the host antibody response over the course of infection. The depletion of B cells is well-documented in mouse models of C57BL/6 and BALB/c infected with *T*. *b*. *brucei* [30,31] and believed to be an important mechanism of immunosuppression to allow parasite survival.

**Fig 6:**
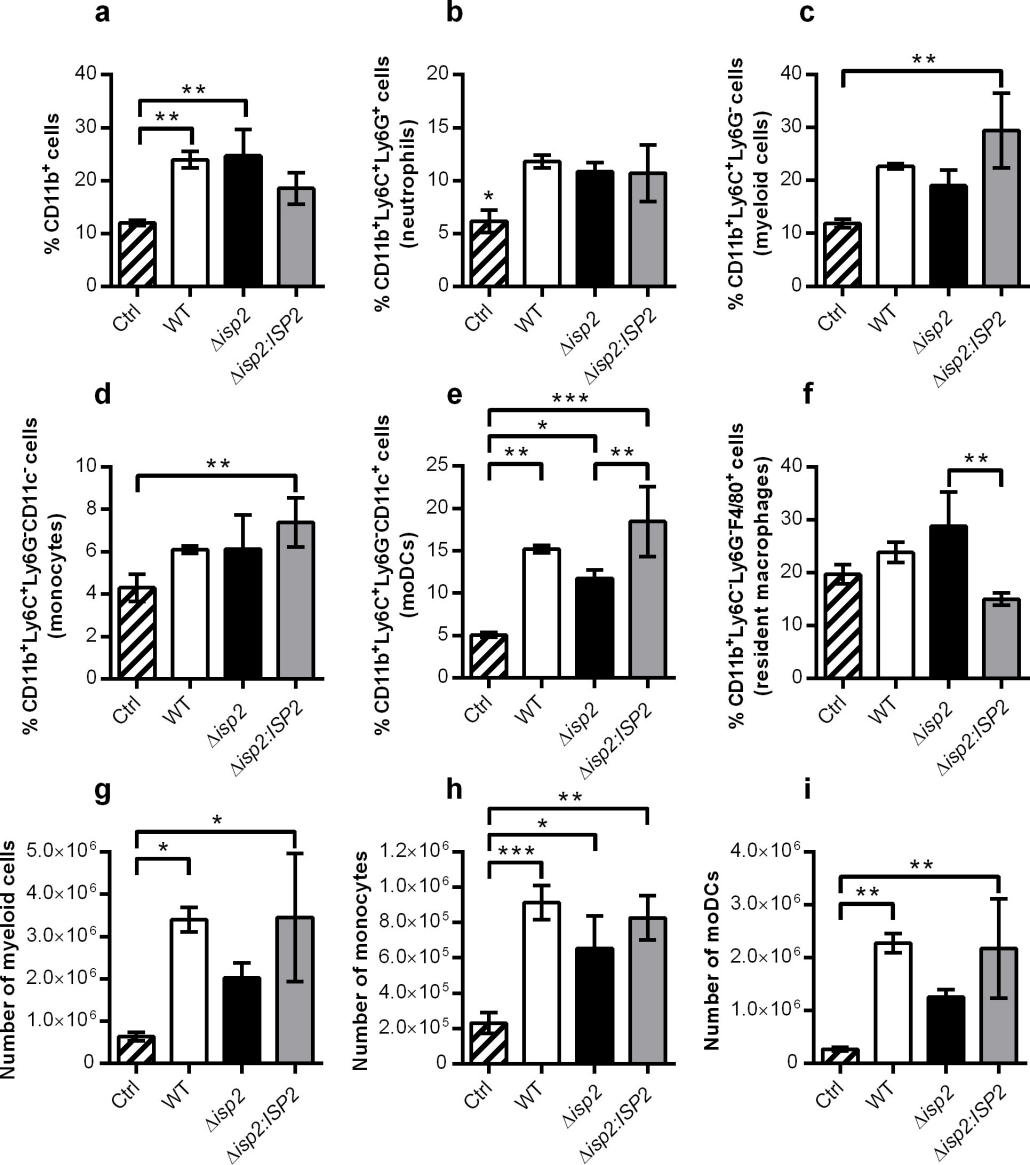
Distribution of innate cellular sub-populations in the spleen during early infection. C57BL/6 mice were infected intraperitoneally with 1×10^5^ *T*. *b*. *rhodesiense* WT, Δ*isp*2 and Δ*isp*2:*ISP*2. Uninfected mice were used as a control (Ctrl). After 4 days, mice were euthanized, perfused, and the spleens were removed and homogenized. Cell suspensions were then stained with antibodies for extracellular markers for flow cytometry to assess cell subsets. Cells were first gated to exclude debris using the FSC vs. SSC plot, followed by FSC-H vs. FSC-A to exclude doublets. CD11b^+^ cells were selected from a SSC vs. PE-CD11b plot. From the CD11b^+^ cells, a BV605-Ly6C vs. APC-Ly6G plot was generated to identify neutrophils (Ly6C^+^Ly6G^+^), resident cells (Ly6C^-^Ly6G^-^) and myeloid (Ly6C^+^Ly6G^-^) cells. From the resident cells, a histogram for FITC-F4/80 was used to identify F4/80^+^ cells in this subset. From the myeloid cells, a further plot using SSC vs. PerCP-Cy5.5-CD11c was used to identify CD11c^-^ and CD11c^+^ cells. Gating strategies are shown in S3 Fig. Graphs show the percentage of (a) CD11b^+^ cells, (b) CD11b^+^Ly6C^+^Ly6G^+^ cells (neutrophils), (c) CD11b^+^Ly6C^+^Ly6G^-^ (myeloid cells), (d) CD11b^+^Ly6C^+^Ly6G^-^CD11c^-^ (monocytes), (e) CD11b^+^Ly6C^+^Ly6G^-^CD11c^+^ (monocyte-derived dendritic cells, moDCs) and (f) CD11b^+^Ly6C^-^Ly6G^-^F4/80^+^ (resident macrophages), within the CD11b^+^ population in the spleen. The number of (g) myeloid cells, (h) monocytes, and (i) monocyte-derived dendritic cells are also given. n = 3 mice per group. Results are presented as the mean ± SD. Data are representative of 3 independent experiments. Statistical analysis was performed using One Way ANOVA with Bonferroni posttest. * p <0.05, ** p <0.01 and *** p <0.001.

Next, we analyzed the subcellular populations associated with innate responses. At day 4, the frequency of CD11b^+^ cells had doubled in infected mice, as compared to uninfected controls (Fig 6A), which is in agreement with the engagement of innate mechanisms during early infection. The frequencies of the cellular subtypes within the CD11b^+^ population were subsequently determined. The proportion of neutrophils (CD11b^+^Ly6C^+^Ly6G^+^) (Fig 6B) and of myeloid cells (CD11b^+^Ly6C^+^Ly6G^-^) (Fig 6C) increased by 100% in infected mice, as compared to control mice. While the percentage of monocytes (CD11b^+^Ly6C^+^Ly6G^-^CD11c^-^) remained similar between infected and control mice (Fig 6D), there was an increase in monocyte-derived dendritic cells (moDCs) (CD11b^+^Ly6C^+^Ly6G^-^CD11c^+^) (Fig 6E) in infected animals as compared to control mice, the latter being compatible with the requirements to build adaptive immunity. However, Δ*isp*2-infected mice displayed lower, and significantly different, frequency of moDCs, as compared to WT and Δ*isp*2:*ISP*2-infected mice, in which it was increased by 3-fold (Fig 6E). The frequency of macrophages (CD11b^+^Ly6C^-^Ly6G^-^F4/80^+^) was virtually unaffected (Fig 6F), with the exception of Δ*isp*2:*ISP*2-infected mice, which showed significant reduction in the percentage of macrophages. The determination of the total cell counts of each cellular subtype revealed that Δ*isp*2-infected mice had reduced numbers of myeloid cells (Fig 6G), monocytes (Fig 6H) and moDCs (Fig 6I) as compared to WT and Δ*isp*2:*ISP*2-infected mice. Taken together, these results suggest that during early infection, Δ*isp2* parasites failed to induce early expansion of lymphocytes (Fig 5), in contrast with WT and Δ*isp*2:*ISP*2-infected mice, accompanied by reduced generation of moDCs (Fig 6), which could influence the onset of the adaptive response at later stages.

**Fig 7:**
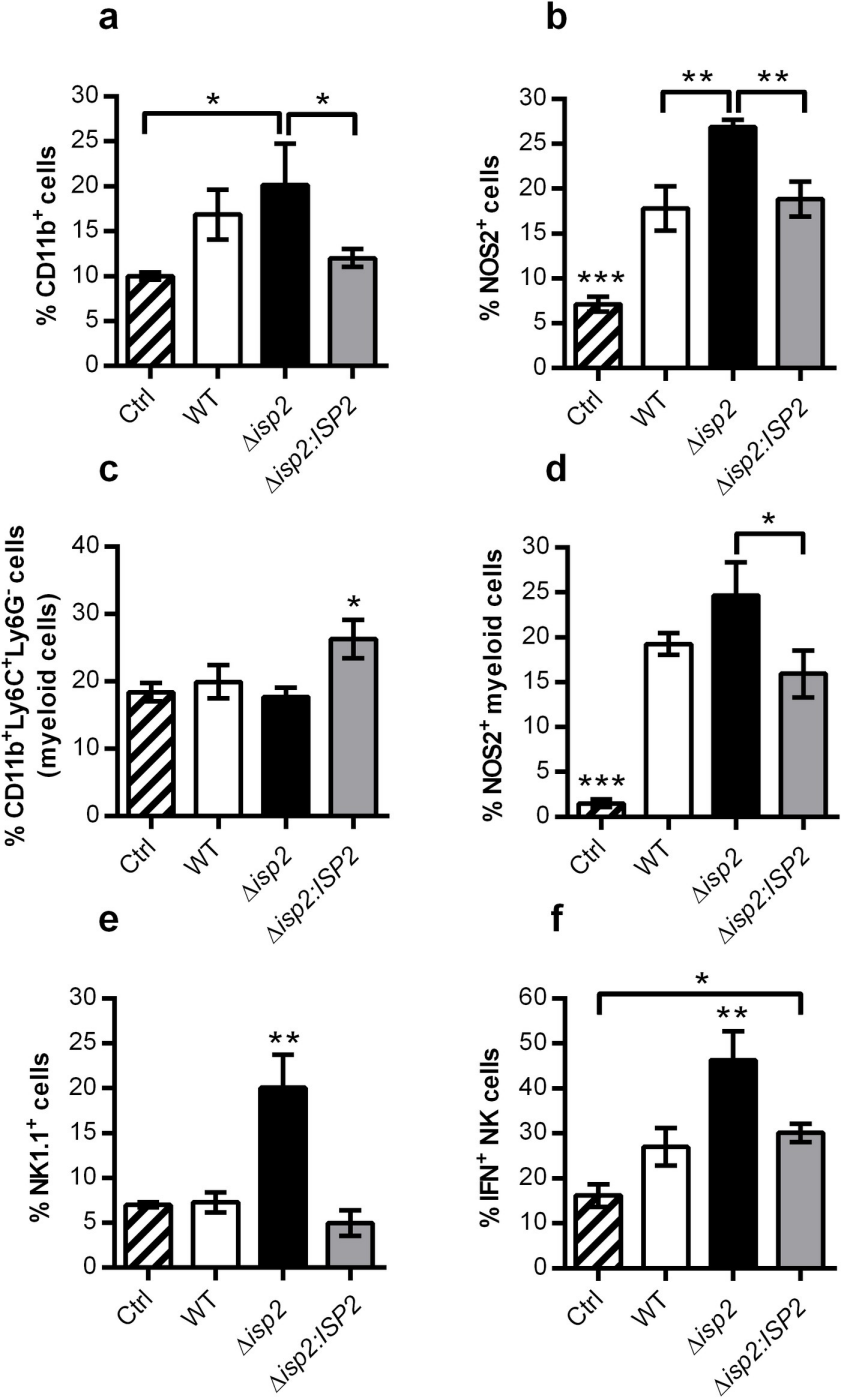
Enhanced NOS2^+^ and IFN-γ^+^ populations in the spleen of mice infected with Δ*isp2 T*. *b*. *rhodesiense* during early infection. C57BL/6 mice were infected intraperitoneally with 1×10^5^ *T*. *b*. *rhodesiense* WT, Δ*isp2* and Δ*isp2*:*ISP2*. Uninfected mice were used as a control (Ctrl). After 4 days, mice were euthanized, perfused, and the spleens were removed and homogenized. Cell suspensions were incubated with monensin for 4 h then stained with antibodies against extracellular and intracellular antigens for flow cytometry to assess the percentage of cell subsets. Cells were first gated to exclude debris using a FSC vs. SSC plot followed by FSC-H vs. FSC-A to exclude doublets. For (a-d) CD11b^+^ cells were selected from a SSC vs. FITC-CD11b plot, then Ly6C^+^Ly6G^-^ (myeloid) cells were identified using a BV605-Ly6C vs. APC-Ly6G plot or gated for the NOS2^+^ cells using a SSC vs. PE-NOS2 plot. The Ly6C^+^Ly6G^-^ cells were further assessed for NOS2 expression using a histogram. For (e and f) total IFN-γ^+^ cells were selected using a SSC vs. APC-IFN-γ plot, or NK1.1^+^ cells were selected using a SSC vs. BV421-NK1.1 plot then further assessed for IFN-γ expression using a histogram. Gating strategies are given in S4 and S5 Figs. Graphs show the percentage of (a) CD11b^+^ cells, (b) NOS2^+^ cells in the CD11b^+^ population, (c) CD11b^+^Ly6C^+^Ly6G^-^ (myeloid cells) within the CD11b^+^ population, (d) NOS2^+^ myeloid cells, (e) NK1.1^+^ cells (natural killer cells, NK cells) and (f) IFN-γ^+^ NK cells. n = 3 mice per group. Data are represented as the mean ± SD. Data are representative of 3 independent experiments. Statistical analysis was performed using One Way ANOVA with Bonferroni posttest. * p <0.05, ** p <0.01 and *** p <0.001.

The apparent weaker response at early stages in animals infected with Δ*isp2* parasites was unexpected since those mice were more capable of controlling infection later on. We asked if there were functional differences in the innate cellular subtype populations, and verified two parameters indicative of the inflammatory response associated with *T*. *brucei* infections: iNOS (or NOS2) [32] and IFN-γ [6]. Intracellular staining for CD11b resulted in a slight increase in the frequency of this population in Δ*isp*2-infected mice, as compared to the other mouse groups (Fig 7A). Importantly, the frequency of NOS2^+^ cells within this population was significantly higher in Δ*isp2*-infected mice (Fig 7B), evidencing an increased inflammatory response. We also found that although Δ*isp2*-infected mice had the same frequency of myeloid cells in the spleen as WT or Δ*isp2*:*ISP2*-infected mice (Fig 7C), they displayed an enhanced percentage of NOS2^+^ cells within the myeloid population (Fig 7D), indicating functional differences in the host response to Δ*isp2*. Due to the relative low frequencies of monocytes and moDCs, we were unable to unequivocally determine which cellular subtypes among the Ly6C^+^ cells contribute to the increased NOS2^+^ population. Enhanced inflammatory response in the absence of TbISP2 was also observed when we verified NK cells in the spleen. In Δ*isp2*-infected mice, there was not only a 4-fold increase in the frequency of the NK population, which was absent in the other infected mouse groups (Fig 7E), we also observed a 2-fold higher percentage of IFN-γ^+^ NK cells, as compared to WT and Δ*isp2*:*ISP2*-infected mice (Fig 7F), reinforcing the notion that TbISP2 plays a role in downmodulating the host early inflammatory response.

**Fig 8:**
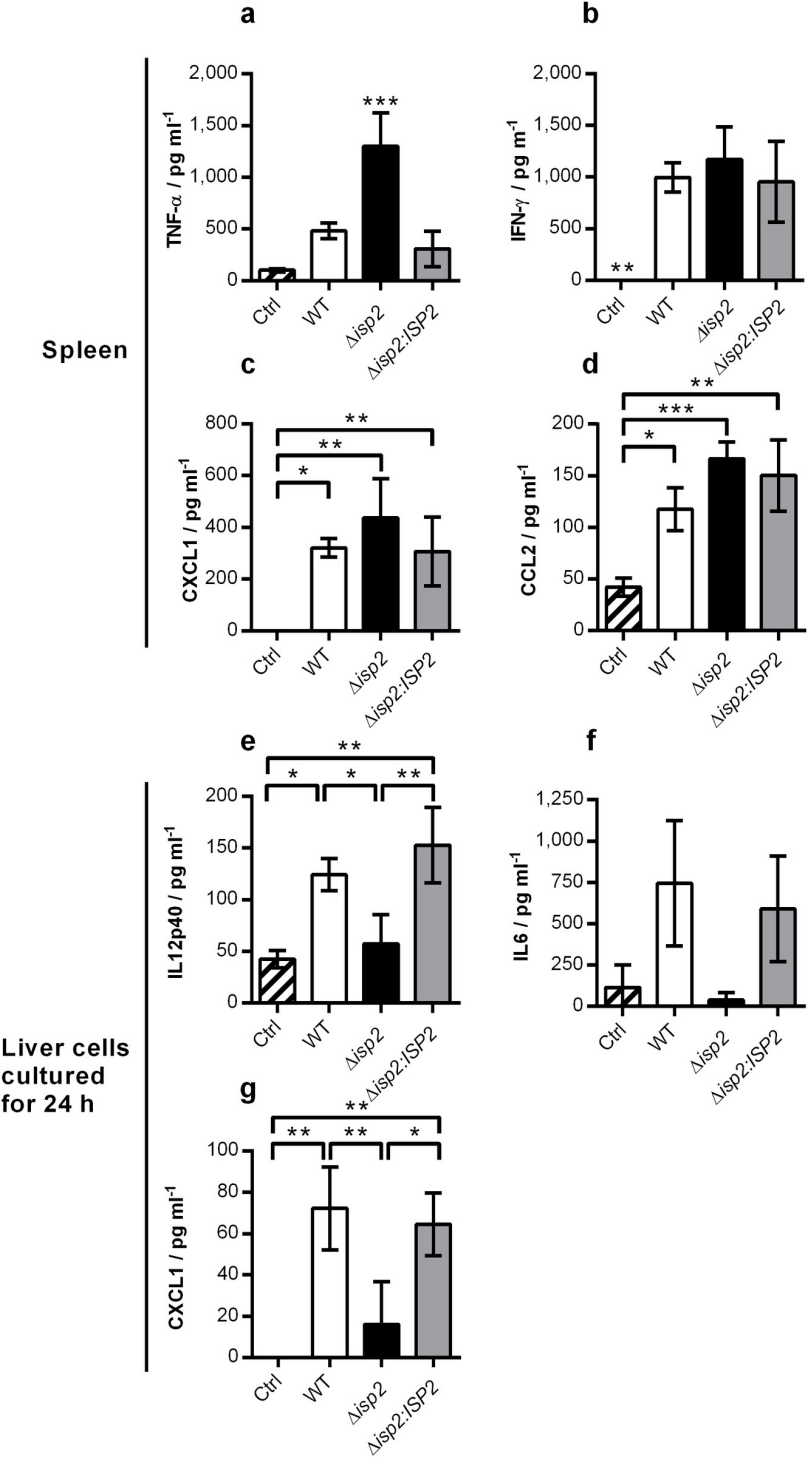
Cytokine levels in spleen and liver of infected mice. C57BL/6 mice were infected intraperitoneally with 1×10^5^ *T*. *b*. *rhodesiense* WT, Δ*isp*2 and Δ*isp*2:*ISP*2. Uninfected mice were used as a control (Ctrl). After 4 days, mice were euthanized and perfused. The spleens and livers were removed and homogenized. Spleens were centrifuged and the supernatants were collected. The concentrations of (a) TNF-α, (b) IFN-γ, (c) CXCL1 and (d) CCL2 were assessed by ELISA. The cell suspensions from the liver were normalized for volume in relation to organ weight and cultured for 24 h at 37°C and 5% CO_2_. Supernatants were then collected and the concentrations of (e) IL-12p40 (n = 3 mice infected with Δ*isp*2), (f) IL-6 and (g) CXCL1 were assessed by ELISA. n = 2 to 3 control mice and 4–5 mice per infected group. Results are presented as the mean ± SD. Data are representative of 3 independent experiments. Statistical analysis was performed using One Way ANOVA with Tukey’s multiple comparison test. * p <0.05, ** p <0.01 and *** p <0.001.

We investigated the response in the spleen further by measuring cytokine levels in the supernatants of tissue macerates after 4 days of infection in mice. We found that TNF-α levels were significantly higher in the spleens of Δ*isp2*-infected mice, as compared to the other mouse groups (Fig 8A), while levels of IFN-γ were equally high in all infected groups (Fig 8B). Chemokines CXCL1 (Fig 8C) and CCL2 (MCP1) (Fig 8D) were also elevated in the spleens of infected animals compared to those of control mice, and there was no significant difference among the infected mice. High levels (>1.5 ng ml^-1^) of IL-12 and CCL5 (RANTES) were similarly detected in the spleen tissues of all infected mouse groups, while the quantifications of IL-6 and CXCL2 were inconclusive. Since the liver was pointed as a key organ in the anti-*T*. *brucei* response [19], we also evaluated cytokine levels in supernatants of cultured liver cells after organ maceration. The pattern observed was inverse to that seen in the spleen, i.e., we found low levels or absence of the inflammatory cytokines, IL-12 (Fig 8E), IL-6 (Fig 8F) and CXCL1 (Fig 8G) in the livers of Δ*isp2*-infected mice, while they were elevated in the livers of WT and Δ*isp2*:*ISP2*-infected mice. The quantifications of IFN-γ, TNF-α, CXCL2 and CCL2 were inconclusive. Taken together, those results showed that in early infection, mice infected with Δ*isp2* parasites display an increased inflammatory response in the spleen, associated with higher percentages of inflammatory cells.

**Fig 9:**
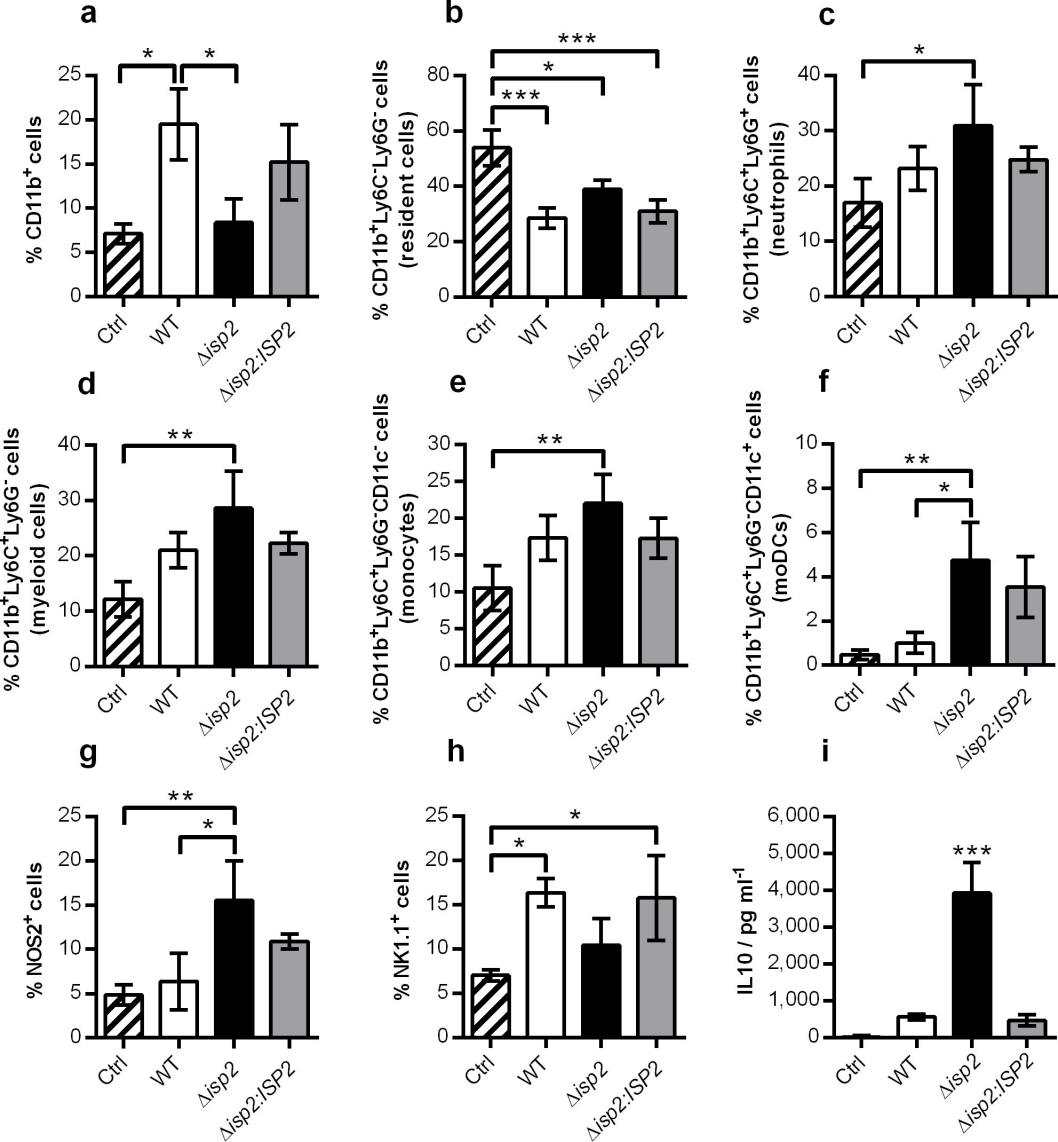
Enhanced NOS2^+^ populations in the spleen of mice infected with Δ*isp2 T*. *b*. *rhodesiense* during late infection. C57BL/6 mice were infected intraperitoneally with 1×10^5^ *T*. *b*. *rhodesiense* WT, Δ*isp2* and Δ*isp2*:*ISP2*. Uninfected mice were used as a control (Ctrl). After 13 days mice were euthanized, perfused, and the spleens were removed and homogenized. Cell suspensions were stained with antibodies for extracellular and intracellular markers for flow cytometry. Graphs show the percentage of cell subsets within the spleen. Cells were first gated to exclude debris using the FSC vs. SSC plot, followed by FSC-H vs. FSC-A to exclude doublets. For (a-f) CD11b^+^ cells were selected from a SSC vs. PE-CD11b plot. From the CD11b^+^ cells, a BV605-Ly6C vs. APC-Ly6G plot was generated to identify neutrophils (Ly6C^+^Ly6G^+^), resident cells (Ly6C^-^Ly6G^-^) and myeloid (Ly6C^+^Ly6G^-^) cells. From the myeloid cells, a further plot using SSC vs. PerCP-Cy5.5-CD11c was used to identify CD11c^-^ and CD11c^+^ cells. For (g) CD11b^+^ cells selected as described above were gated for the NOS2^+^ cells using a SSC vs. PE-NOS2 plot. For (h) NK1.1^+^ cells were selected using a SSC vs. BV421-NK1.1. Gating strategies are given in S4 and S5 Figs. Graphs show the percentage of (a) CD11b^+^ cells, (b) CD11b^+^Ly6C^-^Ly6G^-^ cells (resident cells), (c) CD11b^+^Ly6C^+^Ly6G^+^ cells (neutrophils), (d) CD11b^+^Ly6C^+^Ly6G^-^ (myeloid cells), (e) CD11b^+^Ly6C^+^Ly6G^-^CD11c^-^ (monocytes), (f) CD11b^+^Ly6C^+^Ly6G^-^CD11c^+^ (monocyte-derived dendritic cells, moDCs), (g) NOS2^+^ cells within the CD11b^+^ population and (h) NK1.1^+^ cells (natural killer cells, NK cells). In (i), spleens were centrifuged and the supernatants were collected. IL10 levels in the pooled samples of mice of the same group were determined by ELISA. n = 3 mice per group and n = 4 mice infected with Δ*isp*2. Results are presented as the mean ± SD. Data are representative of 3 independent experiments. Statistical analysis was performed using One Way ANOVA with Bonferroni posttest. * p <0.05, ** p <0.01 and *** p <0.001.

At day 13, the frequencies of CD11b^+^ cells were elevated in the spleens of mice infected with either WT or Δ*isp2*:*ISP2*, as compared to uninfected controls (Fig 9A), suggesting the maintenance of an increased population of innate cells observed at day 4. Since the resident component of the CD11b^+^ population was significantly increased in uninfected mice (Fig 9B), we conclude that the enhanced percentage of CD11b^+^ cells in infected animals originate from recruited cells. In contrast, Δ*isp2*-infected mice showed the opposite profile, in which the frequency of CD11b^+^ cells had returned to basal level, close to that of uninfected controls (Fig 9A). Nevertheless, Δ*isp2*-infected mice still displayed increased frequencies of neutrophils (Fig 9C) and of myeloid cells (Fig 9D), the latter represented by increases in both the monocyte (Fig 9E) and moDC (Fig 9F) subsets. At day 13, there was a functionally distinct response to Δ*isp2* parasites in comparison to the other infected groups, evidenced by increased percentage of NOS2^+^ cells (Fig 9G). In contrast, the frequencies of NK cells had returned to basal levels in mice infected with Δ*isp2*, while it remained elevated in WT and the re-expressor line-infected animals (Fig 9H). Despite, the higher frequency of NK cells in WT-infected animals, the percentage of IFN-γ^+^ NK cells was minimal (<5%) in all infected groups. We detected increased IL10 levels in the supernatant of spleen tissue macerates from mice infected with Δ*isp*2 (Fig 9I), while TNF was undetected. Cytokine determinations in the supernatants of cultured splenocytes were inconclusive. Taken together, the profile of the immune response points to a more prominent early inflammatory response in mice infected with Δ*isp2*, both at the cellular and cytokine level, that develops to a sustained increased population of NOS2^+^ cells later in infection, accompanied by high levels of the anti-inflammatory cytokine IL10.

## Discussion

In this study we show that TbISP2 is expressed by bloodstream form *T*. *b*. *rhodesiense* and is required for sustained virulence in experimental murine infections. We found that ISP2 attenuates the early inflammatory response, thereby contributing to reduce the cellular populations producing IFN-γ and TNF-α, whilst repressing NOS2. ISP2 had been previously shown to play a role in the virulence of *L*. *major* in murine models of infection [25], by downmodulating the infiltration of monocytes and inflammatory response at the lesion site [26]. These studies combine to demonstrate a role for the parasite ISPs in modulating the mammalian host inflammatory response with consequences for parasite survival.

Monoclonal antibodies specific to TbISP2 allowed us to detect it in BSF parasite lysates as a discrete doublet of proteins that migrated at nearly identical molecular mass as recombinant TbISP2. TbISP2 might be susceptible to post-translational modifications that could enhance its molecular mass in SDS-PAGE gels, leading to the recognition of a doublet. In fact, we could predict several potential phosphorylation sites in TbISP2 using the NetPhos 3.1software (http://www.cbs.dtu.dk/services/NetPhos/), while no potential N- or GlcNAc O-glycosylation sites could be predicted. However, we do not know if native TbISPs are modified in these ways in the parasite. No phosphopeptides matching TbISP2 were identified in the phosphoproteome of *T*. *brucei* [33], while the acetylation of TbISP2 at Thr2 was identified in the parasite’s proteome [34]. Alternatively, the doublet might result from some degree of proteolytic processing/degradation of TbISP2. The monoclonal antibodies to TbISP2 detected it distributed in punctate structures in the cytoplasm of *T*. *b*. *rhodesiense*. At present, we cannot precisely determine the identity of those subcellular structures. However, we could not detect ISP2 protein in the parasite flagellum by immunofluorescence, which is in contrast to a previous report that identified TbISP2 in the flagellar proteome of *T*. *b*. *brucei* [35]. ISP2 was also localized to the paraflagellar rod and cytoplasm of *T*. *b*. *brucei* in the TrypTag project (http://tryptag.org/ [36]). It is possible that in the flagellum, TbISP2 is complexed with other proteins, thus making the epitopes that the monoclonal antibodies recognize unavailable for detection here.

Differently from *T*. *b*. *brucei*, which is more commonly used in laboratories to study the biology of African trypanosomes, *T*. *b*. *rhodesiense* is less explored as a model, despite it being one of the subspecies that causes HAT in humans. The *T*. *b*. *rhodesiense* isolate (1852) used here was previously characterized for its ability to induce temporary increase in the permeability of a human brain barrier model through the activation of PAR2 receptors [37,38], and it was also reported to invade the brain parenchyma of mice just 5 hours post-infection [39].

We established a C57BL/6 mouse model for infection with *T*. *b*. *rhodesiense* 1852 that displays a detectable clinical sign of disease—motor disability. Albeit the assessment of the encephalitic stage of experimental HAT was limited due to the lack of analyses of the degree of neuroinflammation, brain pathology and parasite penetration in the CNS, the locomotory dysfunction allowed for a read out to address the potential role of ISP2 in disease development. This model reproduced the pattern of waves of parasitemia that occur in human infections, or in experimental infections with pleiomorphic *T*. *b*. *brucei*, presumably reflecting the cycles of exchange of the VSG coat by the parasite, that enables temporary escape of the host immune response [40]. This parasite subspecies also proved deadly for C57BL/6 mice, which succumbed to death nearly two weeks post-infection. We were able to successfully generate null *T*. *b*. *rhodesiense* mutants for TbISP2 and re-expressor lines that were used to address the potential role of this serine peptidase inhibitor in the host-parasite interaction. There were slightly increased levels of TbISP2 in the re-expressor lines, as compared to WT parasites, together with the staining of a lower molecular mass protein, which could be indicative of protein processing. Since TbISP2 likely functions as a dimer, by analogy to ecotin [28], we cannot predict how the unbalanced levels of full-length versus processed proteins would interfere with inhibitor function in the cell. This could partially explain why, in some instances, the re-expressor line did not fully complement the phenotypes observed with the Δ*isp2* mutants.

TbISP2 proved important for parasite virulence in many parameters of infection: i) blood parasitemia, ii) lethality to the host and iii) manifestation of at least one of the clinical signs of the encephalitic stage of the disease. In HAT, the encephalitic stage is characterized by a plethora of symptoms that can vary among individuals, and most are of challenging assessment in animal models, such as irritability, lassitude, psychiatric and behavioral disturbances [1]. Signs of motor dysfunction are often present in HAT and were reported in mice infected with *T*. *b*. *gambiense* or with *T*. *b*. *rhodesiense* [41,42]. Although we have not directly analyzed parasite penetration in the brain nor the extension of neuroinflammation, the appearance of the motor disorders in the infected mice can be considered an indirect sign of neurodegeneration, and therefore, helped to assess the contribution of TbISP2 to pathogenicity. Sustained undetectable blood parasitemia after two weeks indicated a significant loss of parasite fitness in the mammalian host. Even though peripheral parasitemia was efficiently controlled, CNS infection could still take place, albeit likely at lower levels than in WT-infected animals. Lack of TbISP2 did not fully prevent the development of motor disorder, but it was significantly delayed. This could result from a lower parasite load in the brain, either due to delayed parasite penetration or to penetration at lower numbers, to a more efficient control of parasite burden in the CNS, or a combination of both. Studies employing co-culture of mouse primary microglia cells and *T*. *b*. *brucei* evidenced parasite phagocytosis and microglia activation, including increased production of nitric oxide (NO) [43]. Furthermore, removal of parasites by microglia-mediated phagocytosis was implicated in parasite clearance from the neuropil. It is possible that increased microglial activation in the brain of Δ*isp2*-infected mice had a positive impact in the control of parasite burden, helping to attenuate the motor disorder.

We observed increased levels of CXCL1, CCL2 and TNF-α in the spleens of Δ*isp2*-infected mice after 4 days of infection. CXCL1 and CCL2 were increased in all infected mice as compared to control mice. Macrophages and mast cells are found as the primary sources of the neutrophil chemoattractants, CXCL1 and CXCL2, in the peritoneal wall, the site of parasite inoculation used in this study, and act co-ordinately to recruit neutrophils into injured tissues [44]. Mast cells also release the serine peptidases tryptase and chymase, among others [45], which are potential candidates for ISP2 targets, and that can generate potent chemoattractants *in vivo*. The possible influence of ISP2 in the mast cell proteolytic competence is subject of future investigation.

The onset of the host immune response is considered crucial in determining the extent of pathology in *T*. *brucei* infections, since immunosuppressed mice show increased CNS invasion by the parasite [8]. Inflammatory responses often play an ambiguous role, with IFN-γ production being associated at the same time with the control of blood parasitemia but also with increased parasite and CD4^+^ T lymphocyte penetration in the brain [6]. Elevated levels of IFN-γ were detected in the spleen of all infected mice at day 4, therefore, it is unlikely to have contributed to the reduced blood parasitemia or to the attenuation of motor disorder observed in the Δ*isp2*-infected mice, as compared with WT-infected mice. On the other hand, TNF-α is crucial for parasite clearance, as TNF-knock out (KO) mice have impaired capacity to control the first peak of *T*. *b*. *brucei* in the blood [46]. Conversely, exceedingly high levels of TNF-α might be detrimental to the host, and its contribution to HAT is still controversial [47]. In fact, in a mouse model of infection with *T*. *b*. *brucei*, TNF-α was associated with moderate to severe neuropathology [48] and it can also promote T-cell infiltration in the brain [49]. The 3-fold higher levels of TNF-α found in the spleen of Δ*isp2*-infected mice at day 4, as compared to mice infected with WT or re-expressor lines, could help in the activation of cellular subtypes that subsequently exerted Δ*isp2* clearance from the blood, but at the same time, it could contribute to the onset of disease that includes motor disorders. We were unable to confidently identify the source of TNF-α-producing cells in the spleens of infected mice. In previous work, the levels of systemic TNF-α could be reduced by administration of IL-10 to mice infected with *T*. *b*. *brucei*, which was accompanied by attenuated neuroinflammation and lower CNS parasitosis [50]. At day 13, we detected elevated IL-10 in the spleens of mice infected with Δ*isp*2. It is possible that the delicate balance of pro- versus anti-inflammatory cytokines plays a crucial role in the kinetics of disease progression, taking part in the extended survival and delayed locomotory disorders observed in the Δ*isp2*-infected mice. Anti-parasite responses have been associated with the recruitment of monocytes that can differentiate into macrophages and moDCs, and the accumulation of TNF-induced, iNOS-producing moDCs in the spleen of *T*. *b*. *brucei*-infected mice was shown to actively contribute to tissue damage [51]. The total number and the frequency of moDCs were diminished in Δ*isp2*-infected mice, as compared to WT or re-expressor-infected mice, despite the more elevated TNF-α and NOS expression in the tissue environment, indicating that additional mechanisms, possibly including IL-10, are involved in downmodulating the degree of generation of moDCs.

We found that TbISP2 plays a crucial role in modulating the host immune response to infection in many ways. At day 4 post infection, there were significant increases in the frequencies of cells positive for NOS2 and/or IFN-γ, among the CD11b^+^ and NK populations, respectively, in mice infected with Tb*ISP*2-null mutants. Of relevance, while the frequency of NOS2-expressing cells returned to basal levels in WT and Δ*isp2*:*ISP2*-infected mice, it remained elevated until day 13 in Δ*isp2*-infected mice, indicating sustained competence for NO production. Earlier studies showed that parasitemia is unaffected in iNOS KO mice, suggesting that NO is not critical for parasite clearance [52]. Nevertheless, early decrease in peripheral NO was associated with reduced trypanocidal activity, and can be triggered by the parasite secretome that contains active peptidases capable of inducing a rapid accumulation of asymmetric dimethylarginine, a potent iNOS inhibitor [53], or by parasite kinesin, that steers the cells arginine/NO metabolism towards the production of arginine [18]. We did not evaluate if the BSF secretome is affected in the absence of TbISP2. A report on the secretome of *T*. *b*. *brucei* and *T*. *b*. *gambiense* procyclic forms identified ISP as specifically secreted by *T*. *b*. *gambiense* (referred to as ecotin) [54]. However, the great amount of nuclear and cytoplasmic proteins identified in their secretomes does not allow a definite conclusion on the possible secretion of ISP2. Importantly, it was found that iNOS KO mice have increased parasite and T cell penetration in the brain, as well as elevated cerebral permeability, despite unaffected blood parasitemia [32]. NO has been associated with the prevention of the breakdown on the blood-brain barrier in Wistar rats infected with *T*. *b*. *brucei*, avoiding non-regulated passage of cells, exerting a protective effect for neuroinflammation and brain damage [5]. Taken together, we can hypothesize that in the absence of TbISP2, sustained increased NO helps to keep blood parasitemia under control while preserving the integrity of the blood-brain barrier and protecting the CNS from early parasite and T cell penetration.

Another relevant feature observed in the absence of TbISP2 was the preservation of the T and B lymphocyte populations at day 13. Immunosuppression and B cell depletion and/or loss of B cell memory induced by the parasite are known mechanisms of immune evasion [30,31,55]. NK-mediated cytotoxicity is one of the mechanisms promoting splenic B2-B cell depletion in *T*. *brucei* experimental infections, leading to poor production of parasite-specific antibodies [56]. Noteworthy, the administration of anti-NK1.1 antibodies protected C57BL/6 mice from *T*. *brucei*-induced B cell depletion, showing that in this infection model, NK cells kill B cells and suppress humoral immunity [56]. We observed increased frequency in the NK1.1^+^ population in the Δ*isp2*-infected mice at day 4, which was accompanied by a lower number of B cells. In contrast, the NK1.1^+^ population decreased to nearly basal levels at day 13 in those mice, while it remained elevated in WT or Δ*isp2*:*ISP2*-infected mice. Although not directly demonstrated here, it is possible that, in the absence of TbISP2, the lack of continuous generation of NK cells helped in the preservation of B cells. *T*. *b*. *brucei* VSG mutants with defective removal of host opsonins from the cell surface are more sensitive to phagocytosis by macrophages [57]. In chronic mouse infections, the parasites incapable of removing the anti-VSG antibodies from the surface were less efficient in evading the immune response, leading to parasite clearance [57]. The presumed greater supply of anti-parasite antibodies promoted by increased number and frequency of B cells in the Δ*isp2*-infected mice, could promote parasite clearance by macrophages, contributing to the control of parasitemia.

Altogether, our findings indicate that TbISP2 contributes to the down-modulation of the early inflammatory response by the host, both at the cellular level, by dampening the generation of NO-producing myeloid cells and of IFN-γ-producing NK-cells, and the molecular level, by reducing spleen inflammatory TNF-α. The increased depletion of immune cells, also apparently associated with the presence TbISP2, could ultimately result in the less efficient control of parasite numbers and the development of disease-associated signs. At present, we do not know how TbISP2 exerts its immunomodulatory effect. Although we found it to be a mainly cytoplasmic protein, many parasites are killed during the control of parasitemia waves, and their intracellular contents come readily in contact with the host cells. In *Leishmania* spp., we have identified NE as the main target of ISP2 in macrophages, affecting parasite survival and the control of parasite burden in mice [24–27], but our preliminary analyses did not point to a prominent effect of NE in the *T*. *b*. *rhodesiense* infection model. It remains to be elucidated if additional S1A serine peptidases, such as those belonging to the complement cascade, blood coagulation or present in mast cell or T cell granules undergo modulation by TbISP2, influencing the outcome of anti-parasite immunity and disease pathology.

## Supporting information

S1 Fig Generation of T. *b*. *rhodesiense* 1852 *ISP2*-null mutants. (a) Graphic representation of *ISP2* locus before and after integration of *HYG* and *NEO* resistance cassettes. *Ava*I restriction sites and predicted DNA fragment sizes following enzymatic digestion and probing with 5’ *ISP2* are indicated in the diagram. (b) Genomic DNA was separated on a 0.8% agarose gel and transferred to a nylon membrane before hybridization with 5’ *ISP2*. We detected the fragment corresponding to the endogenous locus (1.35 kb), a 2.6 kb fragment corresponding to the *HYG* allele and a 2.4 kb fragment corresponding to the *NEO* allele. Lane 1, Wild Type *T*. *brucei rhodesiense* IL1852; Lane 2, hygromycin-resistant heterozygote (*HYG*); Lane 3, G418-resitant resistant heterozygote (*NEO*); Lane 4, Δ*isp2* clone 1; Lane 5, Δ*isp2* clone 2. (c) The generation of the *ISP2* re-expressor cell line was confirmed by PCR. Oligonucleotides OL3637 and OL3638 were used to amplify *ISP2* ORF in WT (lane 1), Δ*isp2*:*ISP2* clone 1 (lane 2) and Δ*isp2*:*ISP2* clone 2 (lane 3).
(TIF)

S2 Fig Gating of CD4^+^, CD8^+^ and CD19^+^ lymphocytes. Cells were stained according to **Group 1** in the Methods and were first gated on SSC vs. FSC to exclude debris and then FSC-H vs. FSC-A to exclude doublets. Using a FITC-CD4 vs. PE-Cy5-CD8 plot, CD4^+^ and CD8^+^ T lymphocytes were selected. Using a SSC vs. APC-CD19 plot, CD19^+^ B lymphocytes were selected.
(TIF)

S3 Fig Gating of CD11b^+^ subsets. Cells were stained according to **Group 2** in the Methods and were first gated on SSC vs. FSC to exclude debris and then FSC-H vs. FSC-A to exclude doublets. A plot of SSC vs. PE-CD11b was used to select CD11b^+^ cells, which were then gated based on BV605-Ly6C and APC-Ly6G expression. Ly6C^-^Ly6G^-^ (resident) cells were further gated on FITC-F4/80 expression. Ly6C^+^Ly6G^-^ (myeloid) cells were further gated on PerCP-Cy5.5-CD11c expression. The subpopulations of CD11b^+^ cells were defined as follows: neutrophils, Ly6C^+^Ly6G^+^; resident cells, Ly6C^-^Ly6G^-^; resident macrophages, Ly6C^-^Ly6G^-^F4/80^+^; myeloid cells, Ly6C^+^Ly6G^-^; monocytes (mo), Ly6C^+^Ly6G^-^CD11c^-^; and monocyte-derived dendritic cells (moDC), Ly6C^+^Ly6G^-^CD11c^+^.
(TIF)

S4 Fig Gating of IFN-γ^+^ cells. Cells were stained according to **Group 3** in the Methods and were first gated on SSC vs. FSC to exclude debris and then FSC-H vs. FSC-A to exclude doublets. Cells were selected based on APC-IFN-γ expression for the total IFN-γ^+^ cells, or on BV421-NK1.1 expression for natural killer (NK) cells. NK1.1^+^ cells were further assessed for IFN-γ expression using a histogram.
(TIF)

S5 Fig Gating of NOS2^+^ cells. Cells were stained according to **Group 4** in the Methods and were first gated on SSC vs. FSC to exclude debris and then FSC-H vs. FSC-A to exclude doublets. FITC-CD11b^+^ cells were selected and then gated based on BV605-Ly6C and APC-Ly6G expression, or for the expression of PE-NOS2 within the CD11b^+^ population. Ly6C^+^Ly6G^-^ (myeloid) cells were further assessed for NOS2 expression using a histogram.
(TIF)
