# Supplementary figures and images for: Role of the inhibitor of serine peptidase 2 (ISP2) of Trypanosoma brucei rhodesiense in parasite virulence and modulation of the inflammatory responses of the host
Source: PLoS Negl Trop Dis. 2021 Jun 21;15(6):e0009526. doi: 10.1371/journal.pntd.0009526 (PMC8248637; doi:10.1371/journal.pntd.0009526)

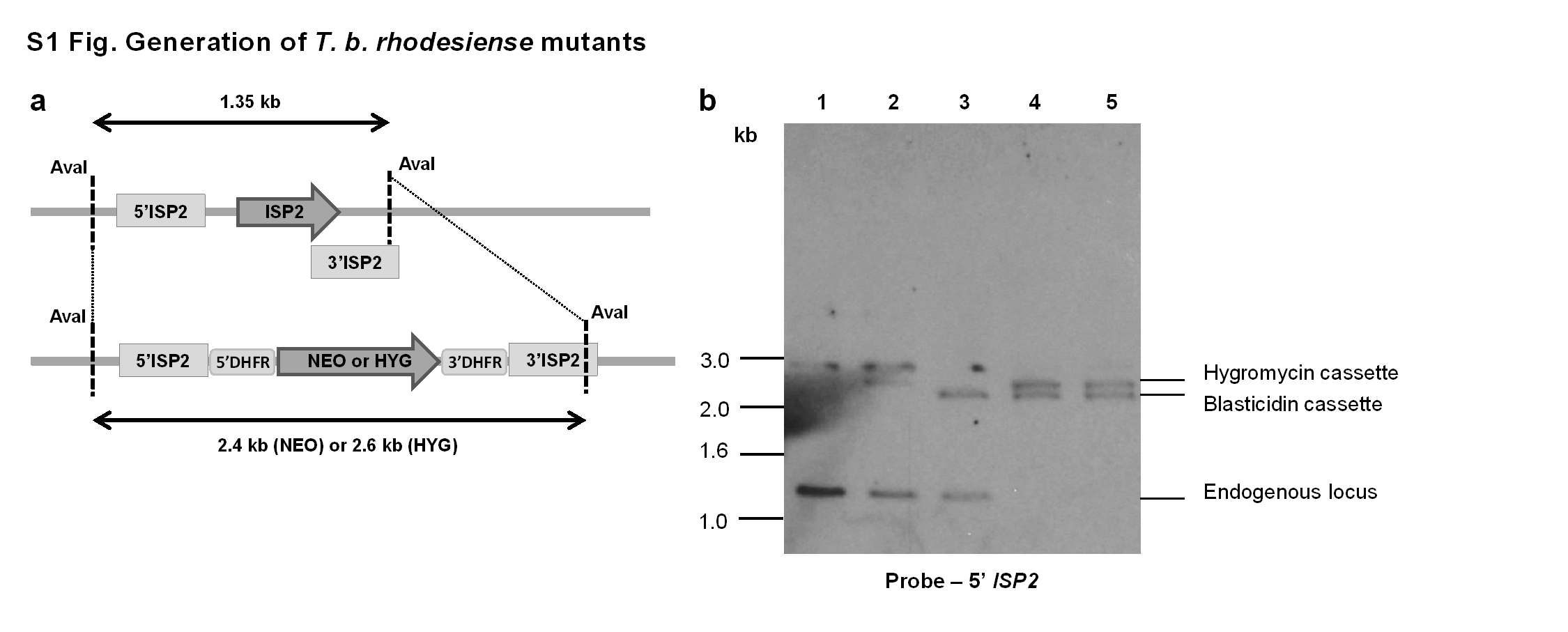

Supplement: S1 Fig — (a) Graphic representation of ISP2 locus before and after integration of HYG and NEO resistance cassettes. AvaI restriction sites and predicted DNA fragment sizes following enzymatic digestion and probing with 5’ ISP2 are indicated in the diagram. (b) Genomic DNA was separated on a 0.8% agarose gel and transferred to a nylon membrane before hybridization with 5’ ISP2. We detected the fragment corresponding to the endogenous locus (1.35 kb), a 2.6 kb fragment corresponding to the HYG allele and a 2.4 kb fragment corresponding to the NEO allele. Lane 1, Wild Type T. brucei rhodesiense IL1852; Lane 2, hygromycin-resistant heterozygote (HYG); Lane 3, G418-resitant resistant heterozygote (NEO); Lane 4, Δisp2 clone 1; Lane 5, Δisp2 clone 2. (c) The generation of the ISP2 re-expressor cell line was confirmed by PCR. Oligonucleotides OL3637 and OL3638 were used to amplify ISP2 ORF in WT (lane 1), Δisp2:ISP2 clone 1 (lane 2) and Δisp2:ISP2 clone 2 (lane 3). (TIF) [file pntd.0009526.s001.tif]

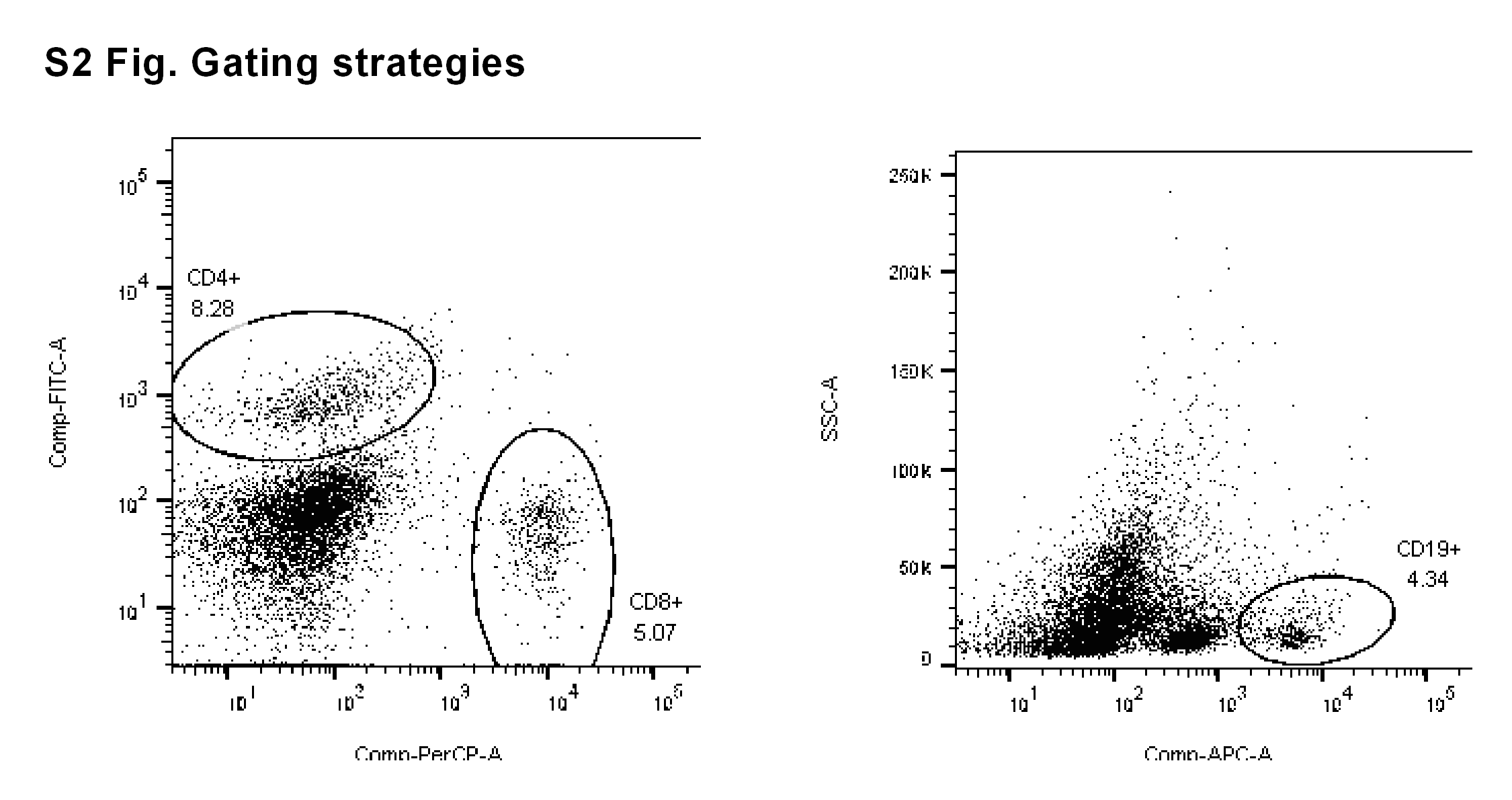

Supplement: S2 Fig — Cells were stained according to Group 1 in the Methods and were first gated on SSC vs. FSC to exclude debris and then FSC-H vs. FSC-A to exclude doublets. Using a FITC-CD4 vs. PE-Cy5-CD8 plot, CD4+ and CD8+ T lymphocytes were selected. Using a SSC vs. APC-CD19 plot, CD19+ B lymphocytes were selected. (TIF) [file pntd.0009526.s002.tif]

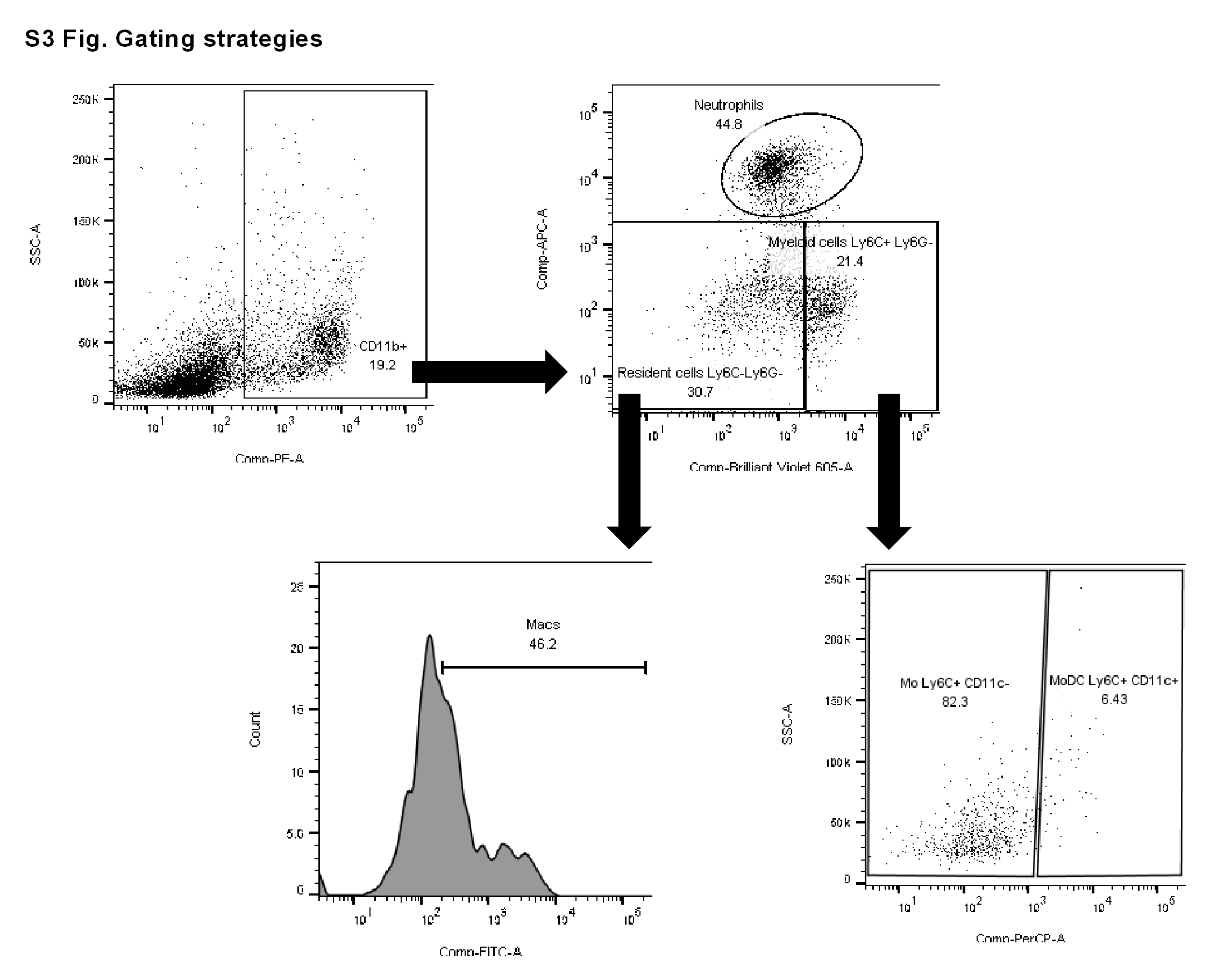

Supplement: S3 Fig — Cells were stained according to Group 2 in the Methods and were first gated on SSC vs. FSC to exclude debris and then FSC-H vs. FSC-A to exclude doublets. A plot of SSC vs. PE-CD11b was used to select CD11b+ cells, which were then gated based on BV605-Ly6C and APC-Ly6G expression. Ly6C-Ly6G- (resident) cells were further gated on FITC-F4/80 expression. Ly6C+Ly6G- (myeloid) cells were further gated on PerCP-Cy5.5-CD11c expression. The subpopulations of CD11b+ cells were defined as follows: neutrophils, Ly6C+Ly6G+; resident cells, Ly6C-Ly6G-; resident macrophages, Ly6C-Ly6G-F4/80+; myeloid cells, Ly6C+Ly6G-; monocytes (mo), Ly6C+Ly6G-CD11c-; and monocyte-derived dendritic cells (moDC), Ly6C+Ly6G-CD11c+. (TIF) [file pntd.0009526.s003.tif]

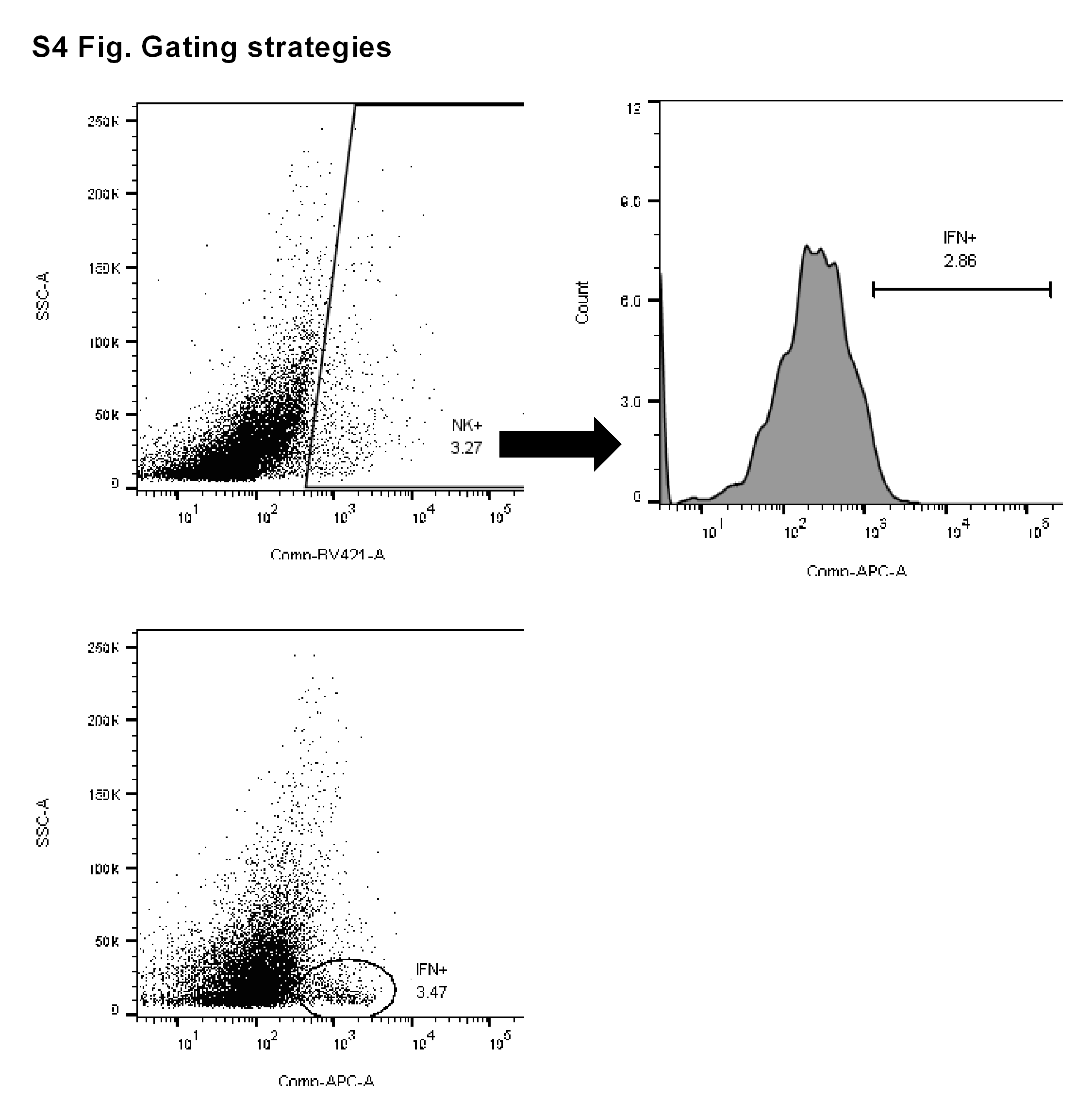

Supplement: S4 Fig — Cells were stained according to Group 3 in the Methods and were first gated on SSC vs. FSC to exclude debris and then FSC-H vs. FSC-A to exclude doublets. Cells were selected based on APC-IFN-γ expression for the total IFN-γ+ cells, or on BV421-NK1.1 expression for natural killer (NK) cells. NK1.1+ cells were further assessed for IFN-γ expression using a histogram. (TIF) [file pntd.0009526.s004.tif]

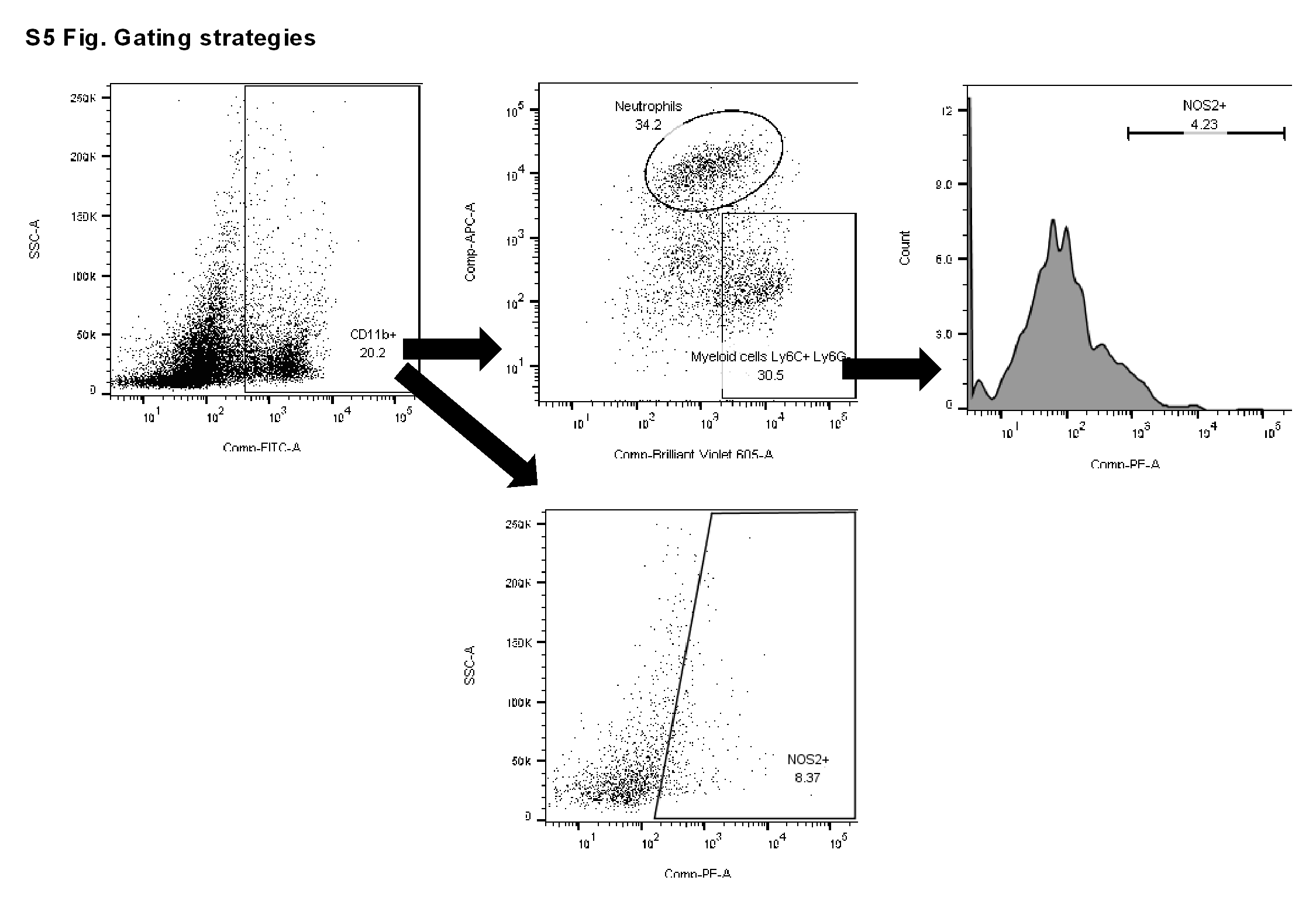

Supplement: S5 Fig — Cells were stained according to Group 4 in the Methods and were first gated on SSC vs. FSC to exclude debris and then FSC-H vs. FSC-A to exclude doublets. FITC-CD11b+ cells were selected and then gated based on BV605-Ly6C and APC-Ly6G expression, or for the expression of PE-NOS2 within the CD11b+ population. Ly6C+Ly6G- (myeloid) cells were further assessed for NOS2 expression using a histogram. (TIF) [file pntd.0009526.s005.tif]
